# Cost-Effective Fiber Optic Solutions for Biosensing

**DOI:** 10.3390/bios12080575

**Published:** 2022-07-28

**Authors:** Cátia Leitão, Sónia O. Pereira, Carlos Marques, Nunzio Cennamo, Luigi Zeni, Madina Shaimerdenova, Takhmina Ayupova, Daniele Tosi

**Affiliations:** 1 i3N, Department of Physics, University of Aveiro, 3810-193 Aveiro, Portugal; sonia.pereira@ua.pt (S.O.P.); carlos.marques@ua.pt (C.M.); 2 Department of Engineering, University of Campania Luigi Vanvitelli, Via Roma 29, 81031 Aversa, Italy; nunzio.cennamo@unicampania.it (N.C.); luigi.zeni@unicampania.it (L.Z.); 3 School of Engineering and Digital Sciences, Nazarbayev University, Nur-Sultan 010000, Kazakhstan; madina.shaimerdenova@nu.edu.kz (M.S.); takhmina.ayupova@nu.edu.kz (T.A.); 4 Laboratory of Biosensors and Bioinstruments, National Laboratory Astana, Nur-Sultan 010000, Kazakhstan

**Keywords:** POC monitoring, smartphone optical biosensors, optical interrogation methods, cancer biomarkers, cardiovascular biomarkers, environmental monitoring

## Abstract

In the last years, optical fiber sensors have proven to be a reliable and versatile biosensing tool. Optical fiber biosensors (OFBs) are analytical devices that use optical fibers as transducers, with the advantages of being easily coated and biofunctionalized, allowing the monitorization of all functionalization and detection in real-time, as well as being small in size and geometrically flexible, thus allowing device miniaturization and portability for point-of-care (POC) testing. Knowing the potential of such biosensing tools, this paper reviews the reported OFBs which are, at the moment, the most cost-effective. Different fiber configurations are highlighted, namely, end-face reflected, unclad, D- and U-shaped, tips, ball resonators, tapered, light-diffusing, and specialty fibers. Packaging techniques to enhance OFBs’ application in the medical field, namely for implementing in subcutaneous, percutaneous, and endoscopic operations as well as in wearable structures, are presented and discussed. Interrogation approaches of OFBs using smartphones’ hardware are a great way to obtain cost-effective sensing approaches. In this review paper, different architectures of such interrogation methods and their respective applications are presented. Finally, the application of OFBs in monitoring three crucial fields of human life and wellbeing are reported: detection of cancer biomarkers, detection of cardiovascular biomarkers, and environmental monitoring.

## 1. Introduction

Biosensors are devices that quantify (or semi-quantify) a biological or chemical analyte by generating a measurable signal proportional to its concentration [1]. They are usually applied in different fields, from biomedical to environmental, allowing monitoring of specific disease biomarkers in body fluids (blood, urine, saliva, and sweat) [2] and detection of micro-organisms [3] and pollutants [4] in the environment, among other things. In the case of optical biosensors, the signal generated can be coded in wavelength, phase, and signal intensity, among other optical features [5]. Optical absorbance, fluorescence, bioluminescence, interferometry, ellipsometry, reflectometric interference spectroscopy, and surface-enhanced Raman scattering are also examples of highly applied optical techniques to biosensing [6].

In relation optical fiber biosensors, usually, the biosensing process relies on the interaction of the evanescent wave with the fiber’s surroundings. Therefore, in order to turn the fiber sensitive to the environmental refractive index (RI) changes, fibers can have gratings inscribed or can be altered in different geometries. Moreover, noble metal coatings with thicknesses around 30–70 nm, enabling the surface plasmon resonance (SPR) phenomena, or nanoparticles, and thus generating a localized SPR (LSPR), can be applied. The use of optical fibers brings important advantages, namely: small dimensions—allowing them to be used in biomedical applications in invasive and non-invasively approaches; dielectric nature—making them electrically safe; and low signal attenuation [7]. The main disadvantages to optical fiber biosensors are the fragility and high-cost interrogation approaches [8]. However, as research on optical fiber sensors (OFS) has been evolving, a large number of studies reporting robust and cost-effective optical fiber biosensing (OFB) approaches has been published in the past few years. The sensing principles that will be described are mainly based on interrogation in the visible spectrum due to the well-known cost-effectiveness of such an approach. However, it should not be forgotten that, in the last few years, efforts that have been made in improving interrogation costs of infrared and gratings spectral analysis. Currently, this is a hot topic in this research field with excellent outputs [9,10,11]. 

**Figure 1:**
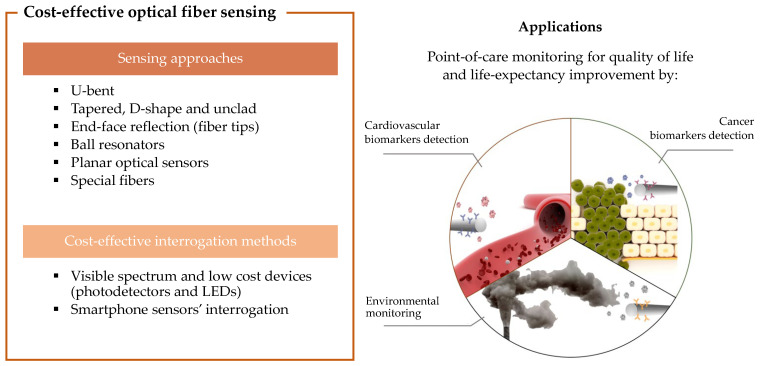
Schematic illustration of the main contents of this review.

This review focuses on the use of OFB approaches that are cost-effective. As can be verified in Figure 1, different fiber geometries and structures will be addressed, namely, U-bent, D-shaped, unclad fibers, tapered fibers, ball resonators, end-face reflection, and light-diffusing fibers (LDFs). Different OFB packaging methods for robustness improvement will be also presented, as well as examples of sensors coupled with smartphones. Cost-effective biofunctionalization methods, mainly based on aptamers and molecularly imprinted polymers (MIPs), will also be discussed. Different applications on cancer and cardiovascular biomarkers’ detection and environmental monitoring will be presented, and finally, a critical analysis of the state-of-the-art and prospects will be made. 

## 2. Cost-Effective Optical Fiber Configurations for Biosensing

Starting from the optical fibers that are usually applied in cost-effective biosensing approaches, different kinds of fibers can be used, from regular silica single-mode fibers (SMFs) [12] to multimode fibers (MMFs) such as plastic-clad silica (PCS) [13] and plastic optical fibers (POFs) [14]. The different kinds of optical fibers, conventional silica-based, microstructured, or specialty fibers in terms of materials and designs offer several useful advantages in developing sensors/biosensors. For instance, when telecommunication wavelength range and setups are used, the silica-based fibers are more appropriate, such as to exploit the monomodal characteristic for several sensing approaches. In contrast, POFs are especially advantageous due to their excellent flexibility, lower attenuation in the visible range, easy handling, multimodal characteristic, and the fact that plastic can withstand smaller bend radii than glass [15].

**Figure 2:**
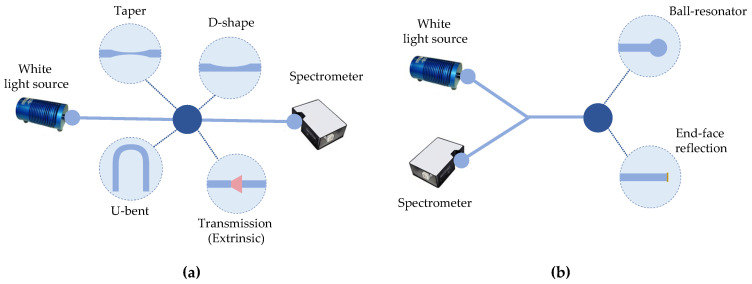
Schematics of OFB working in (**a**) transmission and (**b**) reflection.

As regards the optical signal trajectory, as shown in Figure 2, the different sensing configurations, which will be described as follows, can work on transmission (a) or in reflection (b). Additionally, OFBs can be used as intrinsic and extrinsic OFS. In particular, when the optical fiber interacts with the analyzed medium directly, it is defined as an intrinsic OFS, whereas, when it is used as an optical waveguide allowing the launch of the light to the sensing region and its collection, it is defined as an extrinsic OFS. 

The interrogation methods in the scope of this review are mainly based on the analysis of the visible spectrum, with data codification in wavelength, especially when using SPR and LSPR, and in intensity variations. Although both SMF and MMF can be suitable for wavelength interrogation, as concerns intensity-based approaches, OFBs find great advantage in the use of fibers, having a large core, which implies a high numerical aperture and strongly multimodal operation. The possibility of applying MMFs enables the use of inexpensive light-emitting diodes (LEDs) [16] and simple photodetectors such as charge-coupled devices (CCDs) or photodetectors (PDs) [17], as light can be easily coupled into the fibers using basic optics and cheap interconnections. In contrast, SMFs require hardware with higher complexity and costs.

While the use of a large numerical aperture and strongly multicore fibers is typically a disadvantage in the design of physical sensors, several biosensing structures exploit the favorable properties of MMFs as part of the detection systems. From a light propagation standpoint, in a multimode fiber, modes with a lower index are strongly guided, while higher-order modes are weakly guided and become more sensitive to geometrical modifications of the fiber, such as thinning the fiber core [17], removing the cladding in a D-shaped form [18] or completely, through an etching [19,20] or mechanical [21] process, or bending the fiber in curved shapes [22]. The two most notable approaches for low-cost biosensors based on geometrically modified MMFs are based on U-bent fibers [22,23] and tapered fibers [24]. 

### 2.1. U-Bent Biosensors

**Figure 3:**
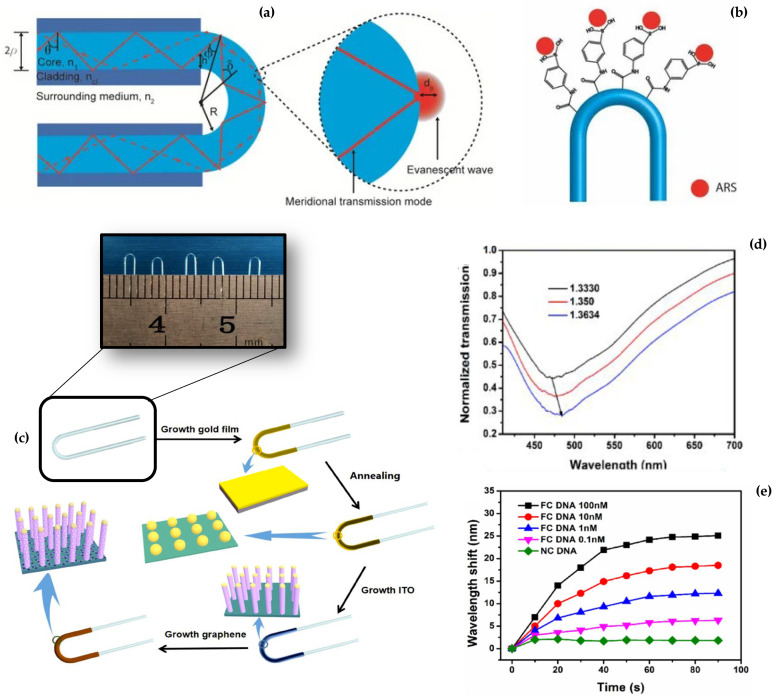
Schematic and implementation of U-shape fiber biosensors: schematic of a U-bent fiber section with light propagation within the curved portion of the fiber (**a**) with inset showing the surface-penetrating meridional rays providing RI sensitivity, and functionalization of the biosensor for glucose detection (**b**) (ARS—Alizarin Red S) (images reproduced from [27]); (**c**) U-bent LSPR sensor proposed by Yang et al. with the respective production steps, namely, U-shape production and photographs, and formation of ITO coating for plasmonic-based sensing, with results of the change in transmission spectrum for three different values of RI (**d**) and the response of the sensor, measuring the wavelength shift over time, for different DNA concentrations (**e**) (images adapted from [28]).

The U-bent fiber is a transmission device operating in the visible spectral range that can be made of a SMF [25] or MMF [15], in which the fiber is curved with a specific bend radius, as highlighted in Figure 3a. During the bending process to obtain the U-shape, the fiber experiences mechanical stress, which causes a variation in its optical properties due to the photoelastic effect. The induced stress will yield a non-uniform RI of the core, with a higher RI at the inner bend and lower at the outer bend of the core. As the bend radius decreases, the RI profile changes more significantly due to the increased stress. Lower order modes are well confined within the fiber and propagate through with minimal losses; higher-order modes, due to the increased angle of incidence at the interface between core and cladding, do not satisfy the total internal condition anymore and are strongly attenuated. Consequently, the intensity of the field increases at the outer region of the core and, at the core-cladding interface, the Fresnel transmission coefficient approximates 1, leading to greater radiation loss. As regards the inner interface of the bent region, more light satisfies the total internal reflection principle, with less light lost in that region. In U-shape fibers, there is a critical radius of curvature, below which the fiber no longer transmits light. The critical radius of curvature decreases for a large difference of RI or large NA values. Larger NA fibers can bend with a smaller bend radius without significant loss of light given the same wavelength of light [26]. 

In order to transform this structure into an efficient RI sensor capable of sensing the surrounding environment, the design [22] makes the use of fiber with an exposed core (hence, with no cladding or depleted cladding) in U-shape. This sensing structure is shown in Figure 3a and was optimized for biosensing according to Azkune et al. [27]. The bent portion of the fiber has no cladding, depleted through wet-etching, dry etching, or by simply fabricating a cladding-less fiber. POF fibers in this regard have been substantially used in U-bent fiber sensors [27,29,30,31] as they are more stretchable, having a lower Young’s modulus, and millions of modes are propagated, hence, guaranteeing a measurable change of transmission when the RI in the surrounding medium changes.

In addition, the U-bent fiber structure shows the best versatility when packaging the device, as the fiber can be tightly folded within a narrow space. Chen et al. [32], among others, reported a U-bent fiber sensor folded in a 1.1 mm tube structure, which is compatible with most medical needles, to fabricate a device with a high level of miniaturization.

The possible functionalization of a U-bent fiber section, as reported in Figure 3b [27], relies on immobilization of bioreceptors on the bent portion of the fiber, in correspondence with the region with the maximum sensitivity. The authors propose a glucose detection system based on the release of ARS (Alizarin Red S) occurring when glucose is bound through competitive binding to phenylboronic acid.

U-bent fibers are often used in conjunction with SPR effects to improve detection. While a U-bent fiber provides a significant transmission change, the wavelength shift observed due to the plasmonic resonance can be detected in a more robust way. This principle was implemented by Yang et al. [28], who reported a DNA biosensor by a thin-film U-bent plasmonic graphene/ITO (indium tin oxide) biosensor. The main achievements reported by Yang et al. are also shown in Figure 3. The Figure 3c shows the graphene/ITO layer deposition around the core-exposed section of a POF, folded around a 1.3 mm bending diameter in the inner region. The transmission spectrum through the bent section of the fiber shows both an intensity change and a wavelength shift due to the combined effect of the fiber losses of meridional rays and localized SPR at the outer interface (Figure 3d. By measuring the wavelength shift, Yang et al. estimated the sensor response to different full complementary DNA and non-complementary DNA at 0.1–100 nM concentrations, as displayed in Figure 3e.

Several biosensors have been reported as U-bent fiber probes based on MMFs and inexpensive spectrometers and hardware based on these principles. George et al. [29] reported an immunobiosensor for the detection of chikungunya (an infectious disease) through the non-structural protein 3 (CHIKV-nsP3) biomarker, achieving a limit-of-detection (LOD) of 0.52 ng/mL. Bandaru et al. [33] reported the production of a plasmonic U-bent fiber biosensor capable of detecting human immunoglobulin G with ultra-low LOD (∼7 aM). Manoharan et al. [34] described a similar architecture for detecting bacterial endotoxins such as lipopolysaccharides with a LOD of 0.4 ng/mL.

### 2.2. Tapered Fiber Biosensors

**Figure 4:**
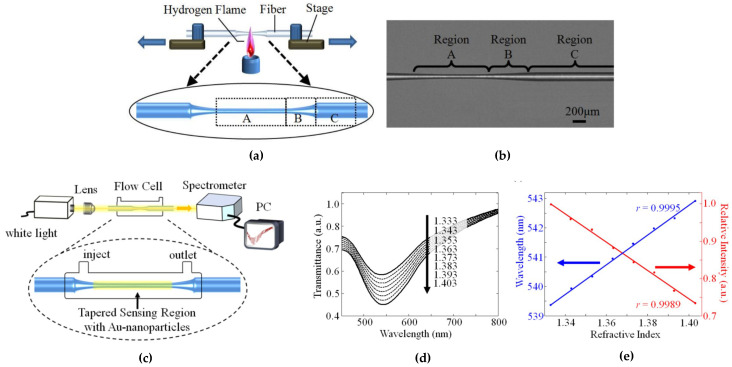
Schematic and implementation of tapered OFB: (**a**) taper production highlighting the respective fiber taper regions and (**b**) optical microscopy image of the tapered fiber showing the three regions; (**c**) taper coated with Au NPs and respective interrogation scheme, and (**d**) results showing the variation in wavelength and transmittance with RI variation with respective linear fittings (**e**) (images reproduced from [39]).

Tapered fibers have been used to implement fiber-optic interferometers [35] or multi-parametric sensors [36] in SMFs. In MMFs, tapers are used to induce losses in the fiber without the need for bending, which allows for easier packaging of the sensor in medical devices [37]. The fabrication of fiber tapers is implemented by modern CO_2_ or fiber laser splicers [38] as a rapid process; it is, therefore, compatible with large volume manufacturing. Figure 4 shows a tapered silica SMF with LSPR for biosensing, developed by C. Huang et al. [39]. As can be seen, in a tapered fiber, the diameter of the fiber is progressively thinned until it reaches a minimum diameter in the so-called waist region, that corresponds to the minimum thickness (region A), with an intermediate region with variable diameter (region B). Unlike the etching process, tapering maintains the proportion between core and cladding diameter. When exposed to a thickness change, the lower radius of the core filters out the higher-order modes as the V number of the fiber decreases, reaching its minimum in the waist region [37]. For the case of an LSPR-based taper, it can be seen in Figure 4d,e that its response changes in wavelength and relative intensity/transmittance in the visible region. 

POF are also good candidates for low-cost tapered fiber structures, as the fiber has very thin cladding and, therefore, allows the core to be exposed to the outer environment. Dash et al. [38] reported a fiber taper in a POF having an initial diameter of 250 μm tapered to 175 μm and coated with a layer of gold nanoparticles (Au NPs) over a 4 mm length. By measuring the output power after the tapered section, a drop is observed when the outer RI increases; a power change of about 5% has been observed when measuring up to 0.2 mM of bovine serum albumin (BSA) concentration. A similar system was reported by Rahman et al. [40] for water salinity detection. 

### 2.3. D-Shaped and Unclad Biosensors

**Figure 5:**
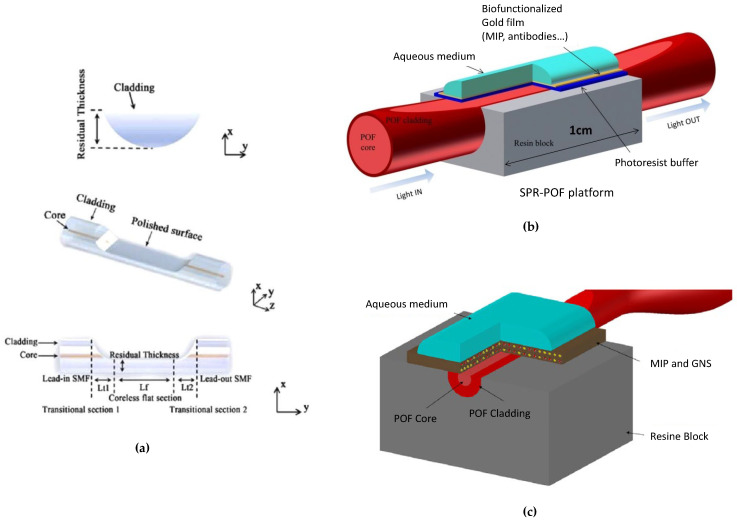
Schematics of D-shaped OFBs: (**a**) core-less D-shape in different perspectives (images adapted from [42]); (**b**) SPR-POF D-shaped biosensing platform (image adapted from [46]. Copyright ©2021, with permission from Elsevier); (**c**) LSPR D-shaped tapered-POF biosensor based on gold nanostars (GNS) and MIPs (image reprinted from [47]. Copyright ©2015, with permission from Elsevier).

As U-bent and tapered OF sensing structures, D-shaping or uncladding an optical fiber aims to increase the interaction of the evanescent field with the surrounding medium. The D-shaped OFS, or side-polished, can be made in SMF and MMF, usually by polishing. As can be seen in Figure 5, the polishing is made until the core surface is exposed [41], but, in other cases, the core can be completely removed in that region [42]. The distance from the polished surface to the fiber surface is usually referred to as the residual thickness and is of utmost importance in the strength of the interaction of the evanescent wave fields with the external medium. For this specific sensor, developed by Zheng et al. [42] and represented in Figure 5a, the optimal RI sensitivity was achieved by a residual thickness of 34.09 μm, attaining a value of 10,243 nm/RIU (RI units). In this kind of structure, the surface roughness also seems to have a crucial impact on the sensor’s response [43]. A great feature that D-shaped OFS have is the flat surface that can be achieved, facilitating deposition of different materials [44] and, also, biosensing testing with only a sample drop [45]. 

Cennamo et al. [14,18] have presented SPR sensors produced by side removing the cladding and part of the core of the POF, spinning a thin layer on the exposed core with a RI major of the core, and finally sputtering a gold nano-film. The buffer layer under the gold nano-film improves the performance of the SPR sensor and the gold film’s adhesion on the platform. Figure 5b outlines these SPR–POF platforms, in which the gold layer can be functionalized with different bioreceptors, namely MIPs [48], aptamers [18], and/or antibodies [49]. Moreover, D-shaped tapered-POFs have also been used to accomplish different kinds of plasmonic bio/chemical sensor configurations, as depicted in Figure 5c, for the detection of trinitrotoluene (TNT) [47]. On the other hand, unclad OFBs have the advantage of a higher interaction of the evanescent wave with the surrounding environment. The removal of the cladding can be achieved by polishing using sandpapers in rotative movements [21] or, in the case of silica fibers, by chemical etching [19].

### 2.4. End-Face Reflection/Optical Fiber Tips

Optical fiber tips are sensors that work on reflection, usually by having the fiber end coated with a reflective/plasmonic material such as silver or gold. In its simpler form, fiber tips are flat at the end and are usually created in MMFs, for instance, PCSs [50], which are interrogated using bifurcated optical fibers connected to the sensor by means of a bare fiber terminator, such as the BFT1 from Thorlabs. To produce flat tips, the fibers are usually cleaved by precision cutters, since a flat surface is crucial for assuring the collected signal spectra reflectivity [51]. Nevertheless, a wide range of tip forms and functionalities can be achieved employing different fabrication technologies, such as mechanical processing, chemical etching, laser processing, self-assembly, and chemical or physical vapor deposition processing, as well as material transfer methods [52]. 

**Figure 6:**
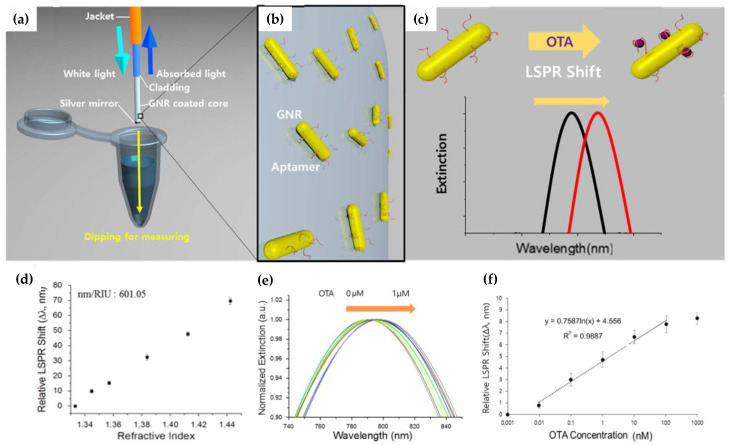
Schematic of a tip OFB: (**a**) components and measurement procedure; (**b**) magnification of the sensor surface biofunctionalized with aptamer-modified GNR; (**c**) schematization of LSPR wavelength shift when the analyte, in this case, OTA, is recognized by the specific aptamer; (**d**) GNR-coated tip OFS response to RI; (**e**) LSPR wavelength shift with different concentrations of OTA, and (**f**) experimental response of the biosensor to different concentrations of OTA with respective linear fitting (images adapted with permission from ref. [53]. Copyright ©2018, with permission from Elsevier).

Fiber tips-based biosensors are expected to be an important tool in POC monitoring due to their high RI sensitivity, compactness, and ease of operation. For example, for flat fiber tips coated with gold by sputtering, it is possible to achieve RI sensitivities between 700 and 2000 nm/RIU depending on the gold film thickness [50]. An alternative approach was reported by Lee et al. [53], who developed a tip aptasensor based on LSPR created with gold nanorods (GNR). The sensor structure and response in the detection of ochratoxin A (OTA) can be seen in Figure 6. 

### 2.5. Ball Resonators

A ball resonator is a device where a circularly shaped microsphere is fabricated on the tip of an optical fiber of approximately a hundred micrometers in diameter. The RI inside the ball resonator is higher than the environment around the spherical structure. In this case, the light that goes through optical fiber and reaches the edges of the microsphere is reflected inside the ball by total internal reflection and, therefore, cannot escape to the surrounding environment [54]. Nevertheless, due to the fact that the surface of the ball resonator has a curved interface, its total internal reflection is never complete; therefore, bending losses could occur, which can lead to the light leakage out of the ball resonator. This leads to an enhanced interaction of samples outside the ball resonator with light thanks to the confinement and rebounding of light inside the circular cavity [55,56]. These evanescent waves are able to interact with both the surface of the microsphere and the environment that surrounds it. Each waveguide of optical field circulation allows it to contact the surrounding environment, which subsequently increases the sensitivity as the amount of circulating rounds rises. The photon lifetime in the microspheric cavity is able to measure it, which is called the quality (Q) factor. The higher the lifetime of the photon, the lower the limit of detection. As previously stated, evanescent waves enable some of the light inside the ball resonator to escape into the outer environment. This allows a microsphere to acquire an increased sensitivity, which makes it useful for a host of applications. Interaction of the surface of the ball resonator with an analyte outside it results in changes in the effective RI. The experience of the analytes of various refractive indexes results in spectral changes such as wavelength and frequency shift. These favorable properties make ball resonators promising as biosensors for the detection of various analytes.

**Figure 7:**
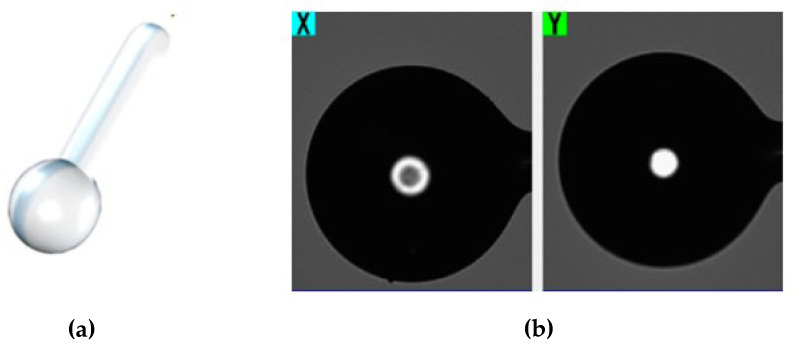
Image of the ball resonator: (**a**) 3D picture of optical ball resonator; (**b**) photographs taken by X and Y cameras of CO_2_ laser splicer. Image reproduced from [57].

The advantage of microspheres is that they can be easily fabricated in the laboratory from various materials, organic and inorganic; they are simple in handling and have a high-quality factor [56]. The ball resonator is a device that requires a quick manufacturing process. A CO_2_ laser splicer allows it to be manufactured in a controllable way in a single step by fusing two single-mode fibers and then tearing them off to make a spherical tip (Figure 7) [57]. Fabrication of ball resonators is advantageous over the fabrication of other optical devices thanks to its duration, which takes from ~60 s up to a couple of minutes depending on the speed of the motors within the machine. Moreover, the fabricated device demonstrates robustness, stability, accuracy in measurements, and higher tensile strength compared to etched fibers, which easily break [57,58,59]. 

In comparison to previously studied optical fiber-based biosensors such as etched Fiber Bragg grating (eFBG), etched tilted Fiber Bragg grating (eTFBG), and etched MgO nanoparticles (NPs) doped fiber, ball resonators show a rather pronounced cost-effectiveness [58,59,60]. The use of a CO_2_ laser splicer enables the achievement of ball resonators with an accurate diameter and low alignment error in a highly repeatable and fast manner within a few seconds. However, there are some limitations in the use of optical ball resonators. Low reflectivity is one of them, requiring the use of an optical backscatter reflectometer which has excellent sensitivity. Moreover, the weak interference phenomenon occurring in the micro-sphere between the multiple reflective paths results in poor visibility of the spectral fringes.

### 2.6. Planar Optical Sensor Chips Monitored via POFs 

POFs can be used to implement simple and highly sensitive optical fiber sensors by exploiting their advantages, such as the excellent flexibility, the easy manipulation and modification, the large diameter, the fact that plastic can withstand smaller bend radii than glass, and the great numerical aperture (NA). In particular, this last characteristic is very useful in comprising extrinsic optical fiber sensor configurations, for instance, for light injection in plasmonic thin bacterial cellulose slab waveguides [61] and InkJet-printed optical waveguides [62].

**Figure 8:**
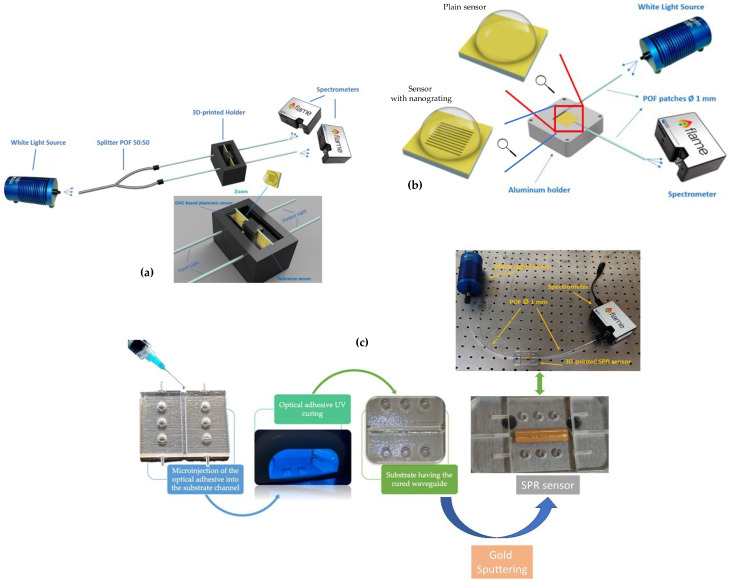
Plasmonic sensor configurations based on extrinsic POF sensors: (**a**) PMMA-based nanoplasmonic sensor chip monitored by exploiting the transparency of the substrate (image adapted from [63]); (**b**) PMMA-based plasmonic chips monitored by a custom setup produced to excite the plasmonic phenomena via the multimode slab waveguide (image adapted from [64]); (**c**) 3D printed SPR sensor chip with the respective setup (image adapted from [62]).

Figure 8 shows several plasmonic schemes based on extrinsic POF sensors. A sensing approach based on a nano-plasmonic sensor chip monitored via a custom 3D-printed holder combined with POFs is reported in Figure 8a. The nano-plasmonic chip is based on a gold nanograting (GNG) fabricated on a poly(methyl methacrylate) (PMMA) substrate by electron beam lithography (EBL) [63]. As shown, the light source is connected through a POF optical coupler (50:50) to two POF patches, one illuminating the sensor with nanograting and the other illuminating the reference sensor (a PMMA chip with the same gold film but without the nanograting). At the output of the holder, there are two POF patches used to collect the transmitted light and send it into two similar spectrometers. In this configuration, the PMMA substrate is considered a transparent substrate.

Alternatively, the PMMA substrate can be regarded as a multimode slab waveguide, as shown in Figure 8b, similar to other plasmonic sensor chips [65,66]. In particular, a gold nano-film can be deposited on the PMMA chip, obtaining an SPR sensor chip [67,68] or producing a gold nanograting on the PMMA chip surface via EBL, generating a nano-plasmonic sensor chip [69]. By using the sensor setup illustrated in Figure 8b, when a gold nano-film is present, the sensor’s performance is similar to that obtained by an SPR D-shaped POF sensor [64], whereas, when a nano-plasmonic chip is used, the orientation of the nanostripes forming the grating pattern, with respect to the direction of the input light (longitudinal or orthogonal), influences the biosensing performances [69].

Figure 8c shows another low-cost 3D printed SPR sensor chip monitored via POFs. This SPR sensor chip has been designed as a disassembled component composed of four different parts by using Autodesk^®^ Fusion 360, and then the STL files were generated [62]. The sensor’s production was performed by a 3D printing method. The material used was a liquid photopolymer ink. Once the SPR sensor parts’ construction was completed, the waveguide core of the 3D printed optical device was fabricated. Thus, a UV photopolymer adhesive was microinjected into the sensor channel and cured for 10 min by means of a lamp bulb with UVA emission at 365 nm. Finally, on the cured core, a gold nanofilm was deposited by a sputtering. The thickness of the sputtered gold was about 60 nm.

### 2.7. Special Fibers

#### LDF-Based SPR Sensors

**Figure 9:**
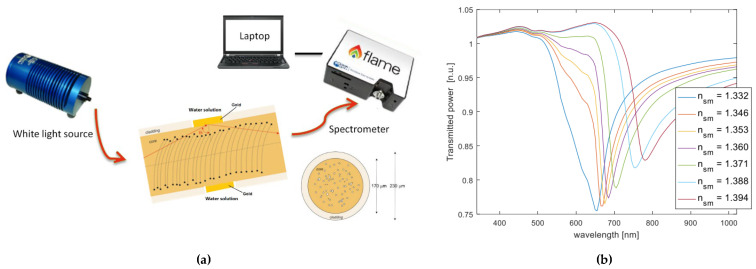
Silica LDF-based SPR sensor system: (**a**) data acquisition and working scheme; (**b**) SPR spectra obtained at different refractive indices from 1.332 to 1.394. Image reproduced from [70].

Recently, an attractive kind of special optical fibers, named Light-Diffusing Fibers (LDF), has been used to generate highly sensitive SPR sensors. In LDF, the light is not confined to the core; on the contrary, it is diffused on the external medium all along their length. Thus, Cennamo et al. have used this aspect to excite the plasmonic phenomenon on metal nano-films deposited on the LDF [70,71,72,73,74]. In particular, this type of SPR sensor can be produced using silica [70,71] or polymer [72] by coating the fiber with metal nano-films, such as gold [70,72] or silver [71], by sputtering. This aspect allows an easier fabrication procedure since only a metal deposition step is required to build the SPR sensor. Moreover, SPR sensors based on silica LDF have already demonstrated high performances in bio/chemical sensing applications, as reported in [73], when the SPR–LDF probe is combined with a bioreceptor (e.g., antibody). For instance, Figure 9a shows an outline of a SPR sensor system based on a silica LDF covered by gold nano-film connected to a white light source and a spectrometer [70]. SPR spectra obtained studying the sensor RI sensitivity, using different concentrations of water–glycerin solutions, are represented in Figure 9b. The experimental results reveal a high sensitivity of the SPR wavelength to the outer medium’s refractive index (bulk sensitivity), with values ranging from 1500 to about 4000 nm/RIU in the analyzed range. Moreover, it was seen in [71] that these bulk sensitivity values can be improved by using silver nano-films instead of the gold. 

Finally, concerning these types of silica SPR–LDF sensors, Cennamo et al. have demonstrated that the tapering process produces a significant worsening of the bulk sensitivity and a slight decrease in the full width at half maximum (FWHM) of the SPR spectra [74]. Therefore, in this SPR–LDF sensor, a possible alternative consists in using the tapered LDF as a modal filter (after the sensitive region) by determining a trade-off between the loss in the sensitivity and the FWHM decrease [74], in a similar way to other configurations, based on different modal filters [75].

Furthermore, low-cost polymeric LDF have been also explored to produce SPR-based sensors [72]. In particular, by sputtering a gold nano-film on the PMMA-based LDF, the obtained bulk sensitivity ranges from 1000 to almost 3000 nm/RIU in the refractive index range from 1.332 to 1.392. In the same work, a novel kind of modal filter has been shown, accomplished by covering the PMMA-based LDF with an aqueous solution to improve the signal-to-noise ratio (SNR) of the SPR sensor, thanks to the filtering of the higher modes, without losing its sensitivity.

## 3. Packaging of Optical Fiber Biosensors

**Figure 10:**
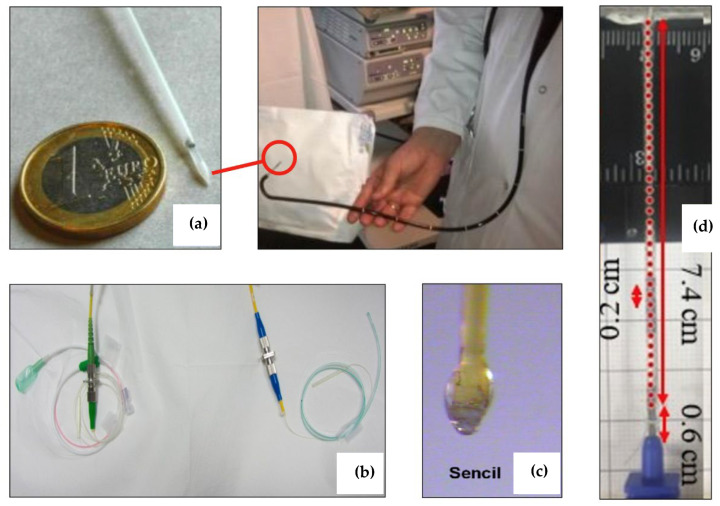
Example of proposed packaging for fiber optic biosensing applications: (**a**) Endoscopic packaging for in situ detection of cancer biomarkers (image reproduced from [78]); (**b**) Urologic packaging, with pressure sensors embodied in a rectal catheter (left fiber) and an abdominal catheter (right fiber) (image reproduced from [79]); (**c**) SencilTM (sensory cilia) packaging for subcutaneous fluorescence sensing (image reproduced from [80]); (**d**) Epidural needle for percutaneous insertion embodying a network of fiber sensors (image reproduced from [81]).

In order to move from the optical biosensing system to practical devices, miniaturization, portability, and easy handling are required. Therefore, strategies for the encapsulation and packaging of the optical components should be considered in order to meet the demands of the final device, namely by preserving the sensing properties. Fiber-optic biosensors are designed to perform real-time detection in medical applications; the possibility to design packaging structures and embed the sensing elements within medical devices allows performing subcutaneous, percutaneous, or endoscopic operations [76]. In addition, wearable technologies designed with optical fiber biosensors embed the fibers within fabric, forming permanent sensing structures [77]. Figure 10 displays some of the options for packaging, targeting different in situ sensing applications.

Endoscopic packaging has been proposed by Guo et al. [78] (Figure 10a) and Loyez et al. [82] for in situ detection of cytokeratin-7 cancer biomarkers. The proposed design integrates the plasmonic biosensor used for detection into a commercially available endoscope that maintains its original functions and allows the precise positioning of the device into the target zone. The work reported in [82] integrates the sensor in a 1.2/1.6 mm (inner/outer diameter) hollow endoscope made with a thermoplastic that maintains a semi-rigid profile. The catheter is terminated by a conical section that allows penetration into the tissue. The active part of the sensor is exposed to the tissue through a fence that allows direct contact between the sensor and its coating and the detection area. The in situ detection reported in this work allows for obtaining discrimination between healthy and tumoral cells.

An example of packaging suited for the urologic diagnostic is shown in Figure 10b [79]; other notable packaging options have been reported by Poeggel et al. [83] and Guo et al. [84]. Urologic diagnostic requires the measurement of biological [84] or biophysical [83] parameters within the bladder and requires additional elements to compensate for external factors, usually requiring a rectal catheter. Urologic devices are flexible silicon rubber hollow catheters that are positioned by the surgeon within the bladder through the urinary tract. The sensors can detect the surrounding environment through a set of side holes in the proximity of the tip.

A form of less invasive packaging, suitable for subcutaneous sensing (underneath the skin), has been reported by Liao et al. in [80] (as shown in Figure 10c) and [85]. The packaging form proposed in these works is labelled as Sencil^TM^, or sensory cilia. The sensor is based on quantum-dot fluorescence made with multimode fiber. The low-cost packaging is based on immersing the sensing tip in a hydrogel: by using a polyethylene glycol acrylate precursor, the fiber forms a thicker tip that can sustain the penetration underneath the skin, reaching a subcutaneous position where the fluorescence-based glucose measurement can be performed.

Percutaneous packaging formats are in use in biomedical applications that require deep-seated diagnostic or therapeutic devices [76]; in this scenario, a rigid needle is used to pierce through the skin for several centimeters, reaching the target area by a straight penetration. Epidural sensors, such as the implementation shown in Figure 10d, proposed by Issatayeva et al. [81], and reported in [86], make use of a Tuohy needle bent around the tip and with fibers mounted on the outer surface.

The packages proposed in [76,77,78,79,80,81,82,83,84,85,86] integrate the sensing tasks with additional purposes, such as delivering a therapy [76], tracing the needle position through X-ray imaging [77], or performing differential diagnostic [83], to form multifunctional, theranostic medical devices.

The integration of sensors in wearable technologies [77], on the other hand, poses the challenge of integrating one or multiple sensors on a device that is worn for a long term. Wearable biosensors based on optical fibers follow two main approaches for fiber packaging [87]. A first method relies on the integration of the fibers into an external element, to be worn separately from the apparel: Issatayeva et al. [81] integrated an array of sensors in two wearable bands, designed to transfer the strain due to respiratory movements to the sensors. 

However, the main trend in wearable devices is to integrate the fibers directly on clothing, where they can continuously monitor vital parameters during sports activities or specific actions. Lo Presti et al. [88] proposed a sensory system based on several gratings integrated into a t-shirt for the measurement of physical parameters during daily activities. Li et al. [89] performed a study of the integration of sensors into textiles by sewing an optical fiber through a piece of fabric and allowing a highly efficient thermal transfer of the body temperature to the sensor. In addition, Esmaeilzadeh et al. [90] reported a biosensor based on the surface plasmon resonance, having the sensing element integrated within a textile for contact sensing.

## 4. Smartphone Fiber Optic Sensors

Smartphones are ubiquitous pocket devices that offer unprecedented diagnostic opportunities for the real-time detection of vital parameters [91]. Modern off-the-shelf phones have several instruments that can be used for sensing purposes [92], and mobile apps can be developed to use each component of the phone to create sensing systems. In addition, apps can use features such as geolocalization, internet of things, or access to external machine learning methods [93] to enhance the potential for diagnostic and biomedical devices.

The potential of smartphones has been exploited within a number of optical sensors [94,95,96,97,98]; the main purpose of the research carried out by several groups in the last few years is driven by replicating diagnostic methods based on fluorescence spectroscopy [94], fluorescence resonance energy transfer [95], colorimetry [96], or spectrophotometry [97], among others, but by replacing the hardware with the existing components on the smartphone, with the minimal addition of external hardware [98].

Overall, optical biosensors based on smartphone platforms are an excellent diagnostic tool and use mounts that can be used as phone accessories in order to integrate the pre-existing devices of the smartphones (such as the flashlight LED or the camera) with additional components such as lenses, spatial filters, diffraction gratings, or additional laser sources [99,100].

Within optical sensors for smartphones, a new generation of optical fiber sensors for smartphones has been developed and reported within the latest years, and it is consistently being applied in the detection of biological analytes, biohazards, and biophysical parameters. While smartphone optical sensors can analyze previously collected samples or can inspect samples positioned in the proximity of the phone’s camera, optical fiber sensors for smartphones introduce the remote sensing possibility. Thanks to the low losses of optical fibers and the availability of multimode fibers that operate in the visible wavelength range, it is possible to design systems that implement the remote sensing feature that is typical of an optical fiber sensor, but where the hardware is partially or entirely replaced by the smartphone itself.

**Figure 11:**
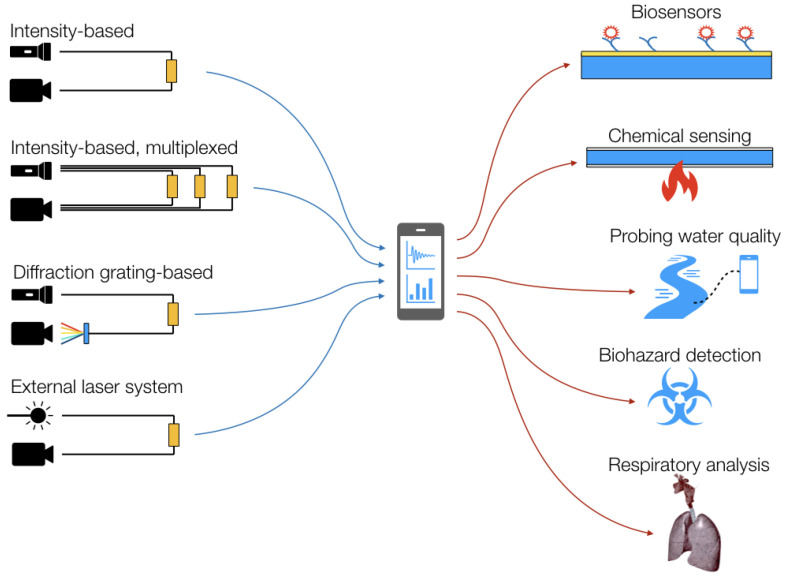
Illustration of the main architectures (**left**) and applications (**right**) of optical fiber biosensors based on smartphones.

Figure 11 illustrates the main implementations and applications of smartphone optical fiber sensors. 

### 4.1. Intensity-Based All-Fiber Smartphone Sensors

Intensity-based sensors represent the simplest implementation, as they can be defined as all-fiber sensors; in these architectures, reported in [101,102,103], the sensing element modulates the light transmitted through the fiber, and both the light source (flashlight LED off) and the light detector (camera) belong to the phone. The only external hardware is a 3D-printed connector that holds the fibers in place and performs light coupling from the flashlight to the input fiber and from the output fiber to the camera.

All systems based on intensity-varying sensors operate with POF fibers, with a core/cladding diameter of 0.98/1 mm and numerical aperture (NA) of 0.47; this wide acceptance angle allows easy coupling of the light in and out of the fibers, delivering a sufficient amount of light to the detector. 

The first system was reported by Sultangazin et al. [101] for biochemical detection of hydrogen sulfide (H_2_S), which is a potentially dangerous biohazard for oil and gas and mining workers; the system uses the flashlight LED from the phone (a phosphor white-light LED illuminating the whole visible wavelength range) and the camera as a pixel-based intensity detector, which converts the output beam from the fiber into greyscale intensity level, and integrates this on the whole set of available pixels. The working principle is based on the change of attenuation in an Ag-coated POF when exposed to hydrogen sulfide; the formation of silver sulfide (Ag_2_S) decreases the wall reflectivity of the outer fiber, increasing evanescent losses and, therefore, reducing the transmission. By integrating, via an app developed in Java, the power incident on the camera, Sultangazin et al. reported an intensity drop of 0.54%/min to 4.9%/min when the sensor is exposed to hydrogen sulfide.

A similar concept was reported by Aitkulov and Tosi [102], extending the sensing mechanism to biophysical sensing by measuring the breathing rate pattern. The sensing element is constituted by a pair of POF fibers, cleaved and spaced by a set distance; when fibers are aligned, they exhibit the maximum transmission, while during breathing movements, the angle between the transmitting and receiving fiber increases the coupling losses, decreasing the detected power. The sensing element is then embodied in an elastic band and worn on the chest for a wearable application. By means of a Fourier analysis of the obtained intensity time signals, the respiratory rate is determined.

The first attempt at a multiplexed fiber-optic sensing system for smartphones was reported in [103]; by merely changing the connector type from the prior system [102], the authors demonstrated a three-fold increase in sensing points. The principle of operation relies on splitting the LED light into multiple fibers and collecting each output into a different portion of the camera. This setup, labelled camera-division multiplexing, allows for isolating each light spot collected on the camera by a tessellation, with minimal crosstalk.

All-smartphone-POF systems [101,102,103] make full use of the phone’s optical circuitry and application programming interfaces for each component. By setting the exposure time, controlled by the ISO of the camera, and disabling the automated routines for the flashlight, it is possible to detect the intensity through photo or video acquisition [102,103] or through a separate mobile app [101]. In this case, no further components are needed to implement the sensing mechanism, as the software can set ISO and calculate the intensity at saturation point, adapting to the power level at the detector. The only external device serves the purpose of mating the phone to fiber and is a phone accessory that can be 3D-printed to be customized to each smartphone model.

### 4.2. Diffraction-Grating Assisted Smartphone Optical Fiber Sensors

The main alternative to power detection via smartphone is to use the camera of the phone as a spectrometer and detect the wavelength shift of spectrally sensitive elements such as surface plasmon resonance (SPR) or fiber Bragg gratings (FBGs). In order to convert the camera to a spectrometer, a diffractive element, such as a grating, or a low-cost element, for instance, a piece of DVD disc, can be used. Thanks to this arrangement, it is then possible to measure the wavelength shift of transmission spectra, making a sensing system that is insensitive to power level and, therefore, potentially more robust to the fiber coupling. 

Bremer et al. [104] presented the first fiber-optic RI sensor adapted for smartphones by means of the SPR effect. The system proposed in this work converts the phone camera to a spectrometer by means of a diffractive element, a holographic grating with 1200 lines/mm mounted at a 45° angle with respect to the camera lens. Then, by detecting the output spectrum sampled by each array of pixels, the spectrum of the SPR sensor was detected, obtaining a sensitivity of 1678 pixel/RIU after optimizing the resolution of the phone’s camera.

The system proposed by Bremer and Roth was adapted in [105] to the detection of SPR in a planar waveguide configuration. Here, the fiber sensor is replaced by a tapered waveguide geometry with an initial thickness of 1200 μm, reduced to 200 μm in the proximity of the sensor. Two SPR sensors have been designed in the waveguide system, enabling multiplexed detection; the waveguide is coated with a thin film of gold in order to immobilize aptamers, which are used as specific bioreceptors for the detection of 25-hydroxyvitamin D. The waveguide system demonstrates the possibility to detect this vitamin D with a device mounted as an external accessory for the phone.

**Figure 12:**
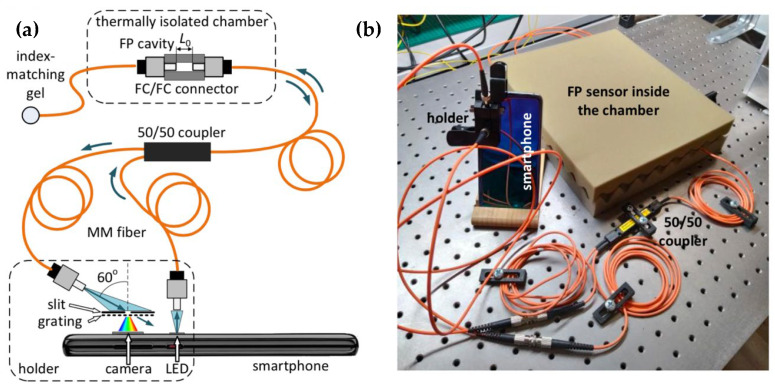
Schematic representation (**a**) and photograph (**b**) of the smartphone optical fiber sensing system for Fabry–Perot sensor interrogation (image reproduced from [106]).

A different approach was proposed by Markvart et al. [11,106], who showed the possibility to interrogate traditional wavelength-selective fiber-optic sensors, such as interferometers [106] or gratings [11], using an architecture based on the smartphone. The first system, shown in Figure 12, is designed for the interrogation of Fabry–Perot (FP) interferometers. The system uses a diffraction element in front of the camera, collecting the light at a 60° angle. The spectrometer is used as a colorimeter through a signal processing method that converts the output image into the corresponding transmission spectrum by means of signal processing.

A similar system was reported in [11] for the interrogation of chirped fiber Bragg grating sensors, inscribed on a multimode fiber for strain and temperature sensing. The chirped grating, with bandwidth ~ 8 nm at the red wavelength, exhibits a wavelength shift that can be detected by estimating the pixel-based spectrum via signal processing. Furthermore, Pan et al. [107] reported a temperature sensor based on a multimode fiber, detected in reflection through a smartphone system similar to the previous ones.

### 4.3. Smartphone Optical Fiber Sensors with External Sources

While the previous architectures makes use of both the light source and detector of the phone, other systems proposed in [107,108,109,110,111] maintain a fiber-optic probe but use a different light source. In these cases, the system is still fiber-coupled and compatible with a smartphone, but significant external hardware is required.

Kamizi et al. [107] developed a fiber-optic sensing network that monitors walking patterns. To do so, a series of single-mode fibers have been placed underneath insoles, with sensors acting as macro-bending units that increase the propagation losses when pressed during the walking motion. The system relies on external hardware that incorporates the LED light source and all the coupling mechanisms, while the output beam is detected through the camera of the phone.

Liu et al. [109] reported a fluorescence fiber-optic sensing system based on a smartphone assisted by an external laser source. The system uses a red laser in order to excite fluorescence in a multimode glass fiber functionalized with quantum dots. The reflected light is collected by the phone’s camera by means of an external module customized for the smartphone’s size and camera position. An Android app is used to calibrate the system and collect the data. Here, the proposed application is the detection of mercury cations (Hg^2+^) in the 1–1000 nM range. A similar system was described in [110] for pH detection and in [111] for dual-channel fluorescence monitoring.

The different main implementation strategies of smartphone-based optical fiber sensors are summarized in Table 1. 

## 5. Biosensing Applications

As introduced before, due to their unique characteristics, OFBs have enormous potential to be used as devices for POC monitoring of biochemical components in diverse fields. This review is focused on three areas that are of utmost importance for the health and wellbeing of the population in general: cancer and cardiovascular biomarkers detection and environmental monitoring.

Real-time, label-free, and high specificity towards a biomarker of interest are a few of the fundamental features of the modern biosensor [6]. Among other essential traits are the miniaturized size of the device and high sensitivity [6]. In the sensing technology, optical fibers demonstrate adaptability for the recognition of numerous analytes and allow the combination of diverse detection techniques. They can monitor refractive index change in the media from basic buffers and water to a complex serum, urine, and blood when detecting proteins, small biomolecules, cells, and other biomarkers [112]. Thanks to strong evanescent fields, optical fiber biosensors modified with biorecognition elements are a promising tool for sensing target molecules [113]. With an increasing demand for enhanced selectivity, specificity, and sensitivity, it is necessary to continue improving optical systems for biosensing applications.

### 5.1. Cancer Biomarkers Detection

Detection of cancer biomarkers plays a significant role in the prognosis and treatment of various cancers [114]. At the early stage of illness, the concentration of biomarkers is extremely low, and this requires highly sensitive diagnostics methods with very low LODs. Although there is ongoing progress in the standard detection and treatment methods, the mortality due to advancement and metastasis of cancer is still high [115]. 

Conventional detection procedures for identifying cancer biomarkers require patients to provide a biopsy of the tumor. Subsequently, the tested sample needs to undergo either fluorescence in situ hybridization (FISH) or immunohistochemistry (IHC) [116,117]. Both testing procedures involve the use of fluorescent tags to label chromosomes or DNA sequences [118] in the former and proteins [119] in the latter case. Since the above testing methods are expensive, take long processing times, and, above all, involve invasive procedures [120], alternative measures need to be called for. The research in the field of cancer detection using optical fiber sensors has made considerable progress where non-invasive, real-time, label-free methodologies are proposed [50,121,122,123,124]. Different optical fiber based systems have been designed and developed to specifically detect cancer biomarkers, aiming towards a fast diagnostic method for POC testing, as will be presented in the following sections. 

#### 5.1.1. Human Epidermal Growth Factor Receptor 2 (HER2)

HER2 (185 kDa) is part of the human epidermal growth factor receptor family, which participates in cell growth and differentiation. However, its overexpression plays an active role in the inception and proliferation of aggressive forms of breast cancer [125]. Patients who undergo costly trastuzumab treatment have a high chance of cardiac toxicity. Thus, taking into account the risk associated with the above therapy, detection of the biomarker is crucial for the prediction and therapy of tumors [126].

In 2017, Sun et al. [124] reported the detection of HER2 on a taper interferometer embedded in FBG. The device was sensitive to RI changes, and wavelength shift was recorded while detecting HER2. The lowest concentration of HER2 detected was at 2 ng/mL. Moreover, the FBG part of the compact device was suggested to be used as an independent thermometer during the sensing process.

An optical fiber-based surface plasmon resonance (OF-SPR) optrode for detection of HER2 was proposed by Loyez et al. [50]. In the experiment, a multimode optical fiber with a diameter of 400 μm was sputtered with gold (thickness 45 nm), followed by silanization and attachment of anti-HER2 aptamers. HER2 biomarkers were detected in phosphate buffered saline (PBS) solution presenting a LOD of 0.6 μg/mL. CK17 protein was tested as a negative control, and no significant shift was observed. Moreover, a sandwich assay experiment using anti-HER2 antibody further improved the LOD to 9.3 ng/mL (~86 pM). 

#### 5.1.2. Cluster of Differentiation 44 (CD44)

CD44 is a transmembrane protein with a molecular weight of 85–200 kDa present in human cells, embryonic stem cells, cancer cells, and cancer stem cells [127]. Its overexpression promotes the migration, proliferation, metastasis, and reoccurrence of cancer [128]. Numerous variant forms of CD44 (CD44v) were confirmed to be related with several malignant tumors and metastasis [129]. Additionally, there is a correlation between the role of CD44 in circulating stem cells and the impact it has on the development of cancer and therapeutic outcome [130].

**Figure 13:**
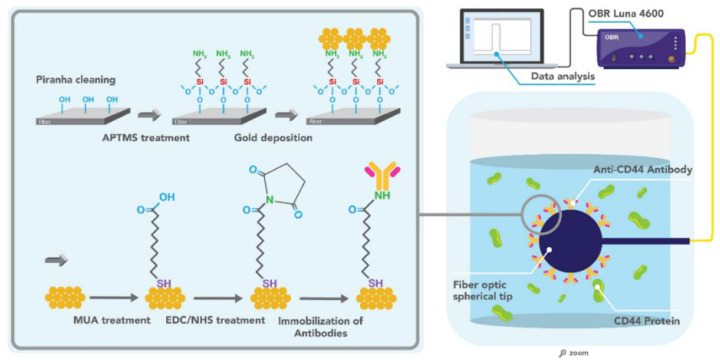
Schematic of functionalization of spherical tip-biosensor used for detection of CD44 biomarker (image reproduced from [131]).

In a recent work from Bekmurzayeva et al. [131], a spherical fiber optic tip fabricated using a CO_2_ laser splicer was investigated. The fiber surface was pre-treated with (3-aminopropyl)trimethoxysilane (APTMS), following several steps to functionalize it with anti-CD44 antibodies, as shown in Figure 13, aiming to detect various levels of CD44. The determined LOD was of 17 pM. In order to demonstrate the specificity of the studies, thrombin and interleukin 4 were used as controls, reporting a minor change in signal. 

#### 5.1.3. Thyroglobulin (Tg)

Differentiated thyroid cancer (DTC) is one of the most common types of endocrine cancer [132]. While the progression rate and death incidence for DTC are indolent, the relapse cases after primary therapy with the need for thyroidectomy are high, which are related to the increased instances of long-term morbidity [133,134,135]. Hence, a lifetime check-up is advised for timely diagnosis to avoid further progression and subsequent death [136]. Thyroglobulin (Tg) is a dimeric precursor glycoprotein weighing 660 kDa, made by the thyroid follicular cells, and is necessary for thyroid hormone synthesis (T_3_ and T_4_). Its levels can be measured in the serum of a healthy person up to 40 ng/mL. The elevated amount of Tg, which is a highly specific biomarker to DTC, is an indication of the latter [137].

In work performed by Kim et al. [138], a fiber-optic LSPR sensor combined with a microfluidic channel was proposed to detect Tg. One of the preparation steps for optical fibers involved treating them with Au NPs (average diameter of 50 nm). The device was exposed to a range of concentrations of Tg, giving the LOD of 93.11 fg/mL. Comparatively, the response time was 10 min, whereas conventional techniques such as chemiluminescence assay (CLIA), immunoenzymometric assay (IEMA), and immunoradiometric assay (IRMA) take a day or more [138].

#### 5.1.4. Cytokeratin 7 (CK7)

There is a discrepancy between the biomarkers expressed by tumors at the early and metastatic stages. Cytokeratin 7 (CK7), which is usually expressed by epithelial cells coating the cavities of internal organs, can assist in the diagnosis of carcinomas. Cancer cells of the lung, breast, thyroid and salivary glands, and female reproductive organs will express CK7. Besides finding the biomarker in primary lung cancer, fragments of cytokeratin can be released into the bloodstream by malignant cells [139].

Ribaut et al. [123], detected CK7 protein (78 kDa) and CK7 (2.6 kDa) peptide using an SPR optical biosensor based on TFBG. In this work, TFBGs with tilt angles between 7° and 9° were coated with gold (thickness of 50 nm) using a high vacuum sputter followed by surface pre-treatment and immobilization of anti-CK7 antibodies. Detection of both CK7 protein and CK7 peptide attained a LOD of 1 pM in PBS and 0.4 nM in serum, respectively. 

#### 5.1.5. Cytokeratin 17 (CK17)

Nearly a third of cases of cancer mortality worldwide are caused by late diagnosis and aggressive growth of lung cancer [140]. The average span of life after confirmation of disease and its subsequent treatment with chemotherapy is less than a year [141]. To enable early-stage diagnosis and to enhance recovery and survival rate, the identification of biomarkers is highly in demand, since various types of lung cancer require various treatment procedures. Generally, an overexpression of cytokeratins is associated with carcinogenesis [142,143]. Among them, Cytokeratin 17 (CK17) promotes cancer progression and epithelial proliferation [144].

Ribaut et al. [121], in 2017, reported a gold-coated (50 nm) TFBG immunosensor with 7° for detection of the CK17 biomarker. The sensor surface was functionalized with a self-assembled monolayer of S_2_-PEG_6_-COOH followed by immobilization of the anti-CK17 antibody. Furthermore, the sensor was packaged into a hollow cylindrical needle, and a window was cut with a laser to expose the fiber to the surrounding environment. The packaged biosensor was used to detect biomarkers in PBS and CK17-encapsulated gel matrix, giving a LOD of 1 pM and 0.1 ng/mL, respectively. In addition, the recognition of analyte was conducted ex vivo in healthy and tumoral tissue samples. Compared to healthy tissue, the sensor’s response to protein showed a linear trend in a biopsy sample of CK17+ positive patients. 

Similarly, Loyez et al. [122] detected CK17 comparing four biofunctionalized TFBG based sensors (without gold—using silanization, gold-coated via sputtering, gold-coated with electroless deposition (ELP), and hybrid gold-coated—a mix of sputtering and ELP) that underwent various pre-treatment procedures. Sensing of analyte showed a linear trend in all four TFBGs. Fiber prepared using silanization showed the lowest sensitivity comparing to the others. However, it was stated that it could be used without taking into account polarization effects. The LOD for the gold-sputtered TFBG was close to 1 pg/mL, and it was the most sensitive device. The sensor was further inserted in the tumorous part of the lung biopsy, showing a positive trend for CK17. Fibers were gold-coated using ELP and hybrid methods and were both sensitive to the analyte, with the former showing sensitivity down to 1ng/mL and the latter being as sensitive as the gold-sputtered TFBG. 

### 5.2. Cardiovascular Biomarkers Detection

Cardiovascular diseases (CVDs) are the prime cause of death in the world. It is estimated that, in 2019, more than 19 million people died from CVDs, corresponding to 32% of all global deaths [145]. Most CVDs are related to lifestyle and modifiable factors [146]. Therefore, prevention is the greatest weapon for reducing CVDs and improving wellbeing. Factors such as stress, sedentary lifestyle, obesity, metabolic diseases, and hypertension are preponderant in the development of such epidemy [147]. Hence, their monitoring and control play a fundamental role in the decreasing of CVDs. Population-level interventions always have to consider the cost-effectiveness of the applied measures and monitoring techniques [147]. In a disease with such a large prevalence, this aspect is of utmost importance.

In CVD management, each person is different, and individual assessment should be personalized according to age, co-morbidities, and lifestyle. Moreover, monitoring should be cost-effective and, when possible, at point-of-care [148]. Recently, different cost-effective optical fiber sensors were developed to monitor different cardiovascular biomarkers, namely, blood pressure [149], cholesterol levels [150] and diabetes mellitus (glucose levels) [151,152], heart acute myocardial infarction (AMI) by Troponins quantification [151,153], heart and kidney failure [154], and stress [21]. 

#### 5.2.1. Detection of Cholesterol and Glucose 

Dyslipidemia, which is the presence of abnormal lipidic levels in the blood, and glucose metabolism disorders, such as diabetes, are independent risk factors of CVDs [147]. Therefore, monitoring of such analytes in POC locations is of outmost importance in CVD prevention. Kumar et al. [150] developed a label-free reflectance-based cholesterol biosensor consisting of SMF with a hollow core fiber working in reflection, coated with Au NPs and with diameters of around 11 nm, for sensitivity enhancement using LSPR phenomenon. The biorecognition molecule was cholesterol oxidase enzyme. With this approach, a LOD of 25.5 nM and a sensitivity of 16.15 nm/μM were attained. In 2019, an optical tapered fiber structure coated with Au NPs, working on transmission, was developed [155]. The taper, created on SMF fiber, achieved a minimum diameter of 40 μm, a total length of 4 mm, and a 5 mm transition stretching region on each side of the taper. The biorecognition molecule was glucose oxidase (GOx) enzyme. This sensing approach was studied in a detection range up to 10 mM, achieving a LOD of 322 μM and a sensitivity of 0.93 nm/mM. In 2020, Zheng et al. [156] described a highly-sensitive glucose sensor, based on back-reflection configuration, using plastic cladding fiber of 600 μm. The fiber tip was coated with gold by sputtering and was then biofunctionalized with GOx enzyme. A measurement range of 0–0.5 mg/mL and a resolution of 0.0004 mg/mL were achieved. 

#### 5.2.2. Detection of Acute Myocardial Infarction Biomarkers

Acute myocardial infarction (AMI) refers to the death of myocardial cells due to ischemia or the imbalance between the blood supply and demand within the coronary arteries as a result of an acute thrombotic process [157]. As a life-threatening situation, the fast diagnosis of AMI is vital for the early initiation of appropriate therapeutic measures. The main tools to diagnose AMI are electrocardiography (ECG) and cardiac troponins detection [158]. Cardiac troponins are regulatory proteins specific to the myocardium that are released into the circulation when myocytes are being damaged [157]. There are three kinds of troponins (Tn): I, T, and C, with troponin T and I being cardiac-specific, while troponin C is expressed by both cardiac and skeletal muscle. 

An ultrasensitive label-free optical microfiber coupler biosensor based on interference turning point effect was developed to detect cardiac troponin I. The interrogation setup consisted of a halogen light source, with a microscope objective to focus the light into the fiber. The sensor consisted of a microfiber coupler fixed in a fluid cell integrated into a polydimethylsiloxane (PDMS) chamber for sample solutions delivery. A visible spectrometer was used as a signal analyzer. With this sensing architecture, an RI sensitivity of 91,777.9 nm/RIU was achieved, and after biofunctionalization with anti-cTnI antibodies and testing in cTnI solutions, it reached a LOD of 2 fg/mL [151]. Krupin and Berini [152] developed a long-range surface plasmon–polariton (LRSPP) waveguide biosensor to detect troponin I. The sensors consisted of gold stripes of about 35 nm thickness, embedded in a low-index optical-grade fluoropolymer (CYTOP^TM^) with fluidic channels etched to the surface of the gold strips. LRSPPs were excited by butt-coupling a polarization-maintaining single-mode fiber (PM-SMF) to the input facet. The optical interrogation included an LED peaking at 1310 nm connected to the PM–SMF. The transmitted light through the LRSPP passes to a 25× objective lens and a 50:50 beam splitter. One part of the split beam is sent to an infrared camera for visual monitoring and alignment, and the other part is sent to a photodetector for evaluation of power variations in time during the detection procedure. The gold waveguide was biofunctionalized with anti-cTnI antibody via Protein G and passivated with BSA. The sensor was tested in a direct and sandwich detection mode, reaching a LOD of 430 pg/mL and 28 pg/mL, respectively. 

#### 5.2.3. Heart and Kidney Failure

Heart failure is a cardiovascular disease characterized by a disorder of the heart, that can be structural and/or functional, which can cause increased intracardiac pressure and/or inadequate cardiac output [147]. In heart failure patients, kidney function has to be closely examined, since renal dysfunction due to diuretics, particularly in patients with heart failure, is a common cause of hospitalization [159]. Botewad et al. [154] developed a biosensor for urea detection, a biomarker of kidney function [160]. This study used as transducer a 450 μm diameter core plastic-clad fiber (PCF) uncladded throughout a 2 cm portion. The uncladded region of the fiber was modified with a composite of polyaniline (PANI) with ZnO and biofunctionalized with urease enzyme, which catalyzes the hydrolysis of urea. This intrinsic sensor worked in transmission using a halogen lamp and a spectrophotometer as light source and detector, respectively. When testing in urea solutions, this biosensor reached a LOD of 10 nM.

In 2021, Li et al. [161] reported a biosensing approach comprised by SMFs, a multicore fiber (MCF), and MMF fibers, in the structure SMF–MCF–MMF–SMF, for the detection of creatinine in the human body. This structure, produced by a fusion splicer machine, was etched to a 90 μm diameter and coated with graphene oxide (GO), Au NPs, and molybdenum disulfide NPs (MoS_2_-NPs) and then biofunctionalized with creatinase enzyme. When tested in creatinine solutions, the sensor presented a LOD of 128.4 μM in a linear range of 0–2000 μM.

In heart failure, higher enzymatic activity with maintained oxygen consumption contributes to the physiopathology of myocardial insufficiency and appears to be an indicator of oxidative stress [162]. In 2021, Ortega-Gomez et al. [51] developed a plasmonic tip biosensor for the detection of reduced cytochrome c, which is a multifunctional enzyme with a crucial role in electron transfer in the mitochondrial transport chain. This biosensor consisted of a PCF MMF uncladded tip, that was coated with Au NP biofunctionalized with cytochrome c as the biorecognition molecule, achieving a LOD of 60 nM.

#### 5.2.4. Stress

Stress conditions (depending on the degree, duration, and individual response) often lead to maladaptive physiological responses and are associated with CVDs in their acute and chronic forms [163]. Stress also increases the probability of developing CVD risk factors such as hypertension, diabetes, and obesity [164]. Prolonged high levels of cortisol, known as the stress hormone, are related to physical and psychological disorders [165]. Therefore, the development of an easy and low-cost POC monitoring method to detect this stress biomarker is a current hot topic. In 2021, C. Leitão et al. [21] reported a POF uncladded approach for the detection of cortisol. The biosensor used SPR as a sensitivity enhancer by coating the unclad part of the fiber with a gold/palladium (AuPd) alloy by the sputtering technique. The surface was biofunctionalized with anti-cortisol antibodies using cysteamine as an intermediated linker. The final surface was passivated with BSA. When tested in cortisol solutions of concentrations between 0.005 and 10 ng/mL, the proposed sensor had a total of 15 nm wavelength shift. The attained sensitivity and LOD were 3.56 ± 0.20 nm/(log(ng/mL)) and 1 pg/mL, respectively. In this research, control tests were also performed in a sensor functionalized with antibodies for human chorionic gonadotropin (anti-hCG antibodies) in which the variance of the resonance wavelength was only 1 nm, much lower than the sensor modified with anti-cortisol antibodies.

### 5.3. Environmental Monitoring

Today, more than ever, there is a growing concern about emerging contaminants and pollutants which severely affect the environment and human health. These contaminants and pollutants, which include insecticides, gases, stimulants, antibiotics, anti-inflammatory drugs, and pesticides, among others, are biologically dynamic and highly resistant. As a result, they continue in the environment, instigating harmful effects to non-target organisms and humans, making it imperative to find approaches capable of correctly detecting these substances in order to protect the environment and our lives.

In agricultural and fisheries engineering, a variety of environmental monitoring sensors (quality of air, soil, and water) allow us to understand the environmental impact of agricultural activities and recognize whether the farm conditions are suitable to cultivate. Many studies have focused on the monitoring of farm soil and aquaculture water, since soil fertility is the key to farmers looking for improvement of crop yields and agricultural productivity, and well-controlled fish water tanks are key to the fishery sector. Further, oil/gas industry pieces of equipment (pumps, pipes, and joints, among others) are required to be monitored. In case any of these become damaged due to natural disasters or human intervention, not only are their properties lost but also the surrounding environment can become polluted. The equipment is submerged at the sea bottom or into a downhole, which are high-pressure and environments with corrosive saltwater. In the case of delivery pipes, these are spread over several kilometers and buried underground. This means that oil and gas industries need non-invasive and low-cost sensing solutions to monitor many critical parameters such as gas detection and/or leakage and oil quality, among other things.

#### 5.3.1. Phenolic Compounds

Phenolic compounds are mainly produced by burning wood and coal apart from their existence as sewage and industrial by-products [166]. With water being the most valuable natural resource for mankind, its pollution by organic and inorganic compounds in the current industrial age has been a serious concern and has placed it as one of the world’s leading health risks [167]. Phenol wastewater is one of the industrial sewages which causes harm worldwide, with it also being one of the worst sources of environmental water pollution [168]. Drinking water with extremely high concentrations of phenolic compounds can cause muscle convulsions, difficulty in walking, and even death [169]. These phenolic compounds release toxic gases during combustion that are potentially harmful to human health if absorbed. *p*-Cresol can enter human bodies in different ways as it is used as a flavoring agent in foods and in some traditional medicines and is always present in tea, oil, and tap water [170]. Recently, an optical fiber-based lossy mode resonance sensor built by using NPs of ZnO/MoS_2_ and the MIP technique was used to detect *p*-cresol [171]. To achieve the specificity of the sensor, tyrosinase enzyme is commonly used for *p*-cresol detection [172]. Tyrosinase (polyphenol oxidase) is an enzyme that catalyzes phenol *o*-hydroxylation yielding *o*-diphenol (monophenolase activity) that subsequently oxidized to *o*-quinone [173]. Very recently, Wang et al. [174] reported that a localized plasmon-based sensor was developed for *p*-cresol detection, consisting of a nonadiabatic 40 μm of tapered optical fiber experimentally fabricated and computationally analyzed using the beam propagation method. For performance optimization of the sensor, two probes were proposed, where probe 1 was immobilized with Au NPs and probe 2 was immobilized with the Au NPs along with ZnO NPs. To increase the specificity of the sensor, the probes were functionalized with tyrosinase enzyme. Different solutions of *p*-cresol in the concentration range of 0–1000 μM were prepared in an artificial urine solution for sensing purposes. Different analytes were prepared for selectivity measurement. The linearity range, sensitivity, and LOD of the probe using ZnO NPs were 0–1000 μM, 5.6 nm/mM (accuracy 0.981), and 57.43 μM, respectively, making the overall performance of such a probe much better, due to the inclusion of ZnO NPs, which increases the biocompatibility of the sensor probe.

#### 5.3.2. Phthalate Esters

Phthalate esters, also known as PAEs, are a type of synthetic chemical substance that has been allocated to the list of priority pollutants because of their endocrine-disrupting and toxic effects on the human body. In 2019, Cennamo et al. [175] reported a D-shaped plasmonic optical fiber biosensor to detect the presence of naphthalene in sea water. The D-shaped configuration was obtained by removing the cladding of the POF by a polishing process. In this study, an antibody specific to the naphthalene molecule was designed and produced and, for that, a retro-synthetic chemical strategy was applied to modify the NAPHTA structure in a derivative structure to obtain the anti-NAPHTA antibodies. This modified NAPHTA structure was coupled to a protein carrier and was used for immunization. The capability of the antibody to bind to naphthalene was assessed by enzyme-linked immunosorbent assay (ELISA) tests. Through *N*-ethyl-*N′*-(3-dimethylaminopropyl)carbodiimide (EDC)/*N*-hydroxysuccinimide (NHS) chemistry, the gold surface was derivatized and functionalized with the produced antibody. Subsequently, tests using real matrices of sea water were performed using the produced biosensor. Through the obtained results, it was possible to observe that the POF biosensor was able to sense the presence of naphthalene in a sea water solution with a LOD of 0.76 ng/mL (0.76 ppb), which is lower than the limit value of naphthalene (0.13 μg/mL). In a 2020 study, Lamarca et al. [176] prepared a label-free U-shaped immunosensor for the detection of ciprofloxacin (CIP) in wastewater samples, as CIP is a broadly utilized antibiotic to treat infections and is a common contaminant of wastewater treatment plants. The glass optical fiber surface was functionalized with PANI, followed by immobilization of anti-CIP antibody. CIP could be detected with a LOD of 3.30 × 10^−3^ ng/L, in a linear range between 0.01 ng/L and 10,000 ng/L, and with a quantification limit of 0.01 ng/L. In addition, the immunosensor offered a high average recovery of 91%. Benzo(a)pyrene (B(a)P) is one of the most toxic polycyclic aromatic hydrocarbons and a carcinogen, making monitoring its concentration levels essential for human health and environmental contamination avoidance. To monitor B(a)P contamination levels in the water, Gao et al. [177] proposed an in-line fiber optofluidic immunosensor using a hollow-core fiber with its surface immobilized with antibodies. As such, the immunoreaction between the antibody and the B(a)P molecule induced a significant change in the RI inside the in-line optofluidic channel. The attained results presented a LOD of 1.65 pM and sensitivities of up to 23 pm/pM.

#### 5.3.3. Gases and Volatile Compounds 

There are myriad gases and volatile materials that researchers and engineers are interested in studying for a number of reasons (ranging from safety issues to general analytical analysis) in the oil/gas industry, from which three will be focused on: formaldehyde (CH_2_O), hydrogen sulfide (H_2_S), and carbon dioxide (CO_2_), where the identification of specific molecules in diverse media and under extreme conditions has been carried out. 

González-Vila et al. [178] developed an MIP coating synthesized around a metal-coated optical fiber sensor by an electropolymerization process, working as an electrode, where the oxidation takes place at the surface of the metal. The electrodeposition was figured by the TFBG–SPR sensor and, as a result of the MIP coating, the TFBG-based sensor acquired sensitivity in gaseous atmospheres. A sensitivity of 2.10 pm/ppm when detecting tiny formaldehyde concentrations in the gaseous state was achieved. In addition, the sensor exhibits a selective behavior to this molecule such that the presence of other volatile compounds did not produce a substantial change in the sensor’s response.

H_2_S is usually found in nature by the decomposition of organic materials, especially in oil and gas production chains. It can also be found in mineral environments such as coal and salt deposits, as well as other mineral extractions containing sulfur. Physical, chemical, and biological agents are the main agents responsible for the formation of this compound. Various systems have been developed using optical fiber sensors due to their large advantages. Sultangazin et al. [101] proposed a low-cost H_2_S sensor based on plastic optical fiber functionalized with silver deposition on the fiber’s outer surface. The sensor is integrated with a smartphone used as an interrogator unit, and the response time is just over a few minutes. Ke et al. [179] presented an optical fiber evanescent-wave sensor. The sensing probe is fabricated by etching a standard single-mode fiber, where the transmitted optical power centered at 1631.9 nm is monitored by a power meter. As the H_2_S gas increases, more energy of the evanescent wave is absorbed, leading to a reduction in the optical power. Prado et al. [180] very recently presented a new study that considers the advantages of optical fiber sensors in conjunction with the colorimetric detection abilities of surface plasmon resonance in Au NPs, reporting the development of a H_2_S detector based on optical fiber coated with Au NPs. The proposed configuration and method used make it possible to detect the presence of H_2_S in gaseous systems while operating at room temperature and with important advantages regarding easy production and short response time. The developed detector has the ability to sense H_2_S levels in the range of 0.4 to 2.0 ppm at room temperature. 

CO_2_ is another critical parameter in the oil/gas industry. There are many materials to use with an optical fiber that have a specific reaction with CO_2_. A range of different materials has been reported in the literature over recent years, such as xerogels doped with 1-hydroxy-3,6,8-pyrenetrisulfonic acid trisodium salt (HPTS) [181], also known as pyranine. This CO_2_ sensor is based on pyranine, a pH-sensitive fluorescent indicator dye. In the presence of CO_2_, the dye has an ion transfer that alters the absorption features of pyranine and xerogel at 396 nm and 460 nm, effectively reducing absorption at these wavelengths, causing reactions at room temperature, and having rational response times. Another approach uses oxidation or reduction reactions in which an electron transfer process occurs between a gas and a material. An example of this is the hybrid nickel oxide/reduced graphene oxide (NiO/rGO), which is a structured coating material. The reactionary wavelengths are 670 nm and 771 nm, and it is the combination of the nanostructured material and its chemical composition in the presence of CO_2_ that triggers the reaction, altering the distribution of the radicals within the materials, changing the electron density and, hence, the permittivity. The result is a small but significant change in the emission wavelengths [182]. A redox reaction using single-wall carbon nanotubes in localized surface plasmon structures has been used for CO_2_ detection [183], where the chemical selectivity is conferred in terms of the activation energy, allowing for room temperature operation.

#### 5.3.4. Aquaculture Monitoring

In agricultural and fisheries engineering, many studies have been explored in terms of soil pollution and critical parameters in water fish tanks. When stress is persistent and uncontrollable, it is considered pathological, which can trigger depression and cardiovascular diseases [184], for example, and therefore the development of technology capable of monitoring stress is essential. Stress involves a large number of neuronal circuits and, once it is promoted, leads to the release of glucocorticoids, in particular cortisol [184]. The substantial variation in this hormone occurs due to exposure to psychological, environmental, or emotional stress [185]. As a result, cortisol is one of the most important stress biomarkers.

One area in which stress also presents significant influence is aquaculture; especially, the impact of such a stress hormone is a challenge to be overcome in recirculating aquaculture systems. When small variations in the water composition or quality occur in these systems, stress induction can arise as well as reduced food intake, which leads to reduced fish growth and, consequently, leads to possible mortality when acute or chronic stress is high. For these reasons and others, it is then essential to monitor cortisol in water [186]. In 2020, Sharma et al. [187] simulated an SPR fiber optic immunosensor for cortisol detection at the wavelength of 830 nm. The sensor consisted of an Ag layer with 2D materials, conventional (graphene, tungsten disulfide (WS_2_), and MoS_2_) and transition metal carbides (MXenes: Ti_3_C_2_, Ti_3_C_2_O_2_, Ti_3_C_2_F_2_, and Ti_3_C_2_(OH)_2_), considered one at a time. The sensor that showed a superior balanced set of performance parameters under both modes was the Ti_3_C_2_O_2_-based probe. Through simulation, this probe was able to achieve a LOD of 15.7 fg/mL. The cortisol sensor developed by Leitão et al. [21], described in the last section, also can be applied to monitor cortisol levels in aquaculture water. 

One of the key limiting factors in aquaculture is the presence of ammonia; therefore, its early detection in small concentrations prevents fish mortality and improves the production quality [188]. Yi Zhu et al. [189] reported a combination of optical fibers and tapered optical fibers (SMF–MMF–taper–MMF–taper–SMF) as miniature interferometry-based optical fiber ammonia gas sensors. A range from 0 to 5460 µg/L of ammonia in the gas chamber was performed. The sensing material involved ZnO nanoflowers deposited on the sensing area (middle MMF and tapers), by a drop of a ZnO solution and 6 h drying in a vacuum oven at 60 °C. Ammonia sensitivity performance is compared between ZnO nanoflowers and ZnO nanospheres of around the same size (1 µm), showing sensitivities of 5.75 pm/(µg/L) versus ~2.25 pm/(µg/L), respectively. Shrivastay et al. [190] presented a contemporary approach to design and developed a hypersensitive ammonia gas sensor producing a Mach–Zehnder interferometer (MZI) by an SMF–PCF–SMF fiber substrate to perform the interference by immobilizing PANI@SnO_2_ nanocomposite to achieve sensing. In this case, excitation of core and cladding modes of PCF is achieved using collapse region that is formed at the junction of SMF and PCF specialty fiber, achieving very fast response and recovery times of 7 and 2 s, respectively, which can detect as low as 8.09 ppt (47.59 fM). The reusable probe showed the potential for rapid detection of ultra-trace ammonia with high selectivity and reproducible features. In 2020, Leal-Junior et al. [191] presented a low-cost fiber-optic probe for the early detection of ammonia. The sensor was based on the chemical interaction between the Oxazine 170 perchlorate layer, deposited in an uncladded polymer optical fiber, and the ammonia dissolved in water. In addition, a thin metallic layer (composed of gold and palladium) was deposited in the fiber end facet and acted as a reflector for the optical signals, enabling the use of the proposed sensor in reflection mode. Different configurations of the sensor were tested, where the effects of the PDMS protective layer, thermal treatments, and the use of reflection or transmission modes were compared in the assessment of ammonia concentrations in the range of 100 ppb to 900 ppb. Results showed better performance (as a function of the sensor sensitivity and linearity) of the sensor with the annealing thermal treatment and without the PDMS layer. Then, the proposed fiber-optic probe was applied to the ammonia detection in high-salinity water, and ammonia concentrations as low as 100 ppb were detected.

Another problematic factor is the usage of myriad pesticides and insecticides in agriculture that are dangerously applied to the soil, such as organophosphorus pesticides that are used worldwide for agricultural purposes. Miliutina et al. [192] reported a functional plasmonic sensor aimed at the monitoring of pesticide spreading and determination of their concentration. It utilized the functionalization of a plasmon-supported fiber surface decorated with a metal–organic framework compound with the formula Zn_4_O(BDC)_3_, also known as the MOF-5 layer. The MOF-5 layer provides the extraction of pesticides from the surrounding medium, which causes the shift in the plasmon resonance absorption band position. The created system demonstrated high selectivity and sensitivity towards organophosphorus pesticides. Particularly, the sensors were successfully applied for the detection of fenitrothion and paraoxon. The lower detectable concentration was found to be as small as 1 pM, which makes the proposed sensors comparable to common analytical approaches to pesticides detection. The proposed cost-effective technique allows for simple and straightforward pesticide detection, even in the complex samples, without any sample’s pretreatment step, and it can be easily and scalable transferred to outdoor conditions. In 2019, Kant et al. [193] presented an SPR based fiber-optic sensor for the pesticide fenitrothion utilizing Ta_2_O_5_ nanostructures sequestered onto a rGO matrix. A thin layer of silver was deposited on the unclad core of silica optical fiber for SPR generation and was followed by the deposition of a sensing surface comprising a layer of tantalum oxide NPs sequestered in a nano-scaled matrix of rGO. The sensing mechanism is based on the interaction of fenitrothion with the silver film, which leads to a change in the RI. Characterized by a wavelength interrogation scheme, the fiber-optic sensor exhibited a redshift equaling 56 nm corresponding to fenitrothion concentration in the range from 0.25 to 4 μM, including the blank solution. The spectral sensitivity is 24 nm/μM, the limit of detection is 38 nM, and the response time is as short as 23 s. The sensor is selective, repeatable, and works at ambient temperature.

## 6. Conclusions

This paper presents a review of optical fiber-based cost-effective biosensing platforms, covering different geometries, interrogation techniques, encapsulation methods, and, finally, presenting applications in three crucial fields of sensing for wellbeing: cancer and cardiovascular biomarkers detection and environmental monitoring. 

The development and application of different types of optical fiber biosensing has increasing over the years at an impressive rate, with the demystification of some associated prejudices. In the past, optical fiber biosensing was labeled as high cost, and for this reason was only considered for highly specific applications. However, as fiber optic biosensor technologies have continued to show excellent performance, the instrumentation associated with their interrogation has dropped in cost, and essentially new methods of designing interrogating sensors have been developed. Fiber-optic sensors working in the visible range are naturally low-cost. Furthermore, the possibility to use the instrumentation of a smartphone to perform the readout of these types of sensors, in addition to considerably lowering the system cost, can be the basis for more democratic and accessible self-monitoring.

Fragility was also one of the points associated with fiber optic sensors. However, various encapsulation techniques are currently used to make these sensors highly robust while maintaining or even amplifying their sensitivity. Currently, we are able to obtain sensors with very high performance, which are highly robust and with practical and low-cost reading techniques. 

The potential of all these cost-effective optical fiber sensing techniques reviewed in this article is supported by the numerous applications and excellent results shown in the three monitoring areas addressed. Nonetheless, as in any other biosensing technique, several aspects still need to be improved and worked on, namely, in the enhancement of their repeatability and reproducibility and by developing techniques to enable their reuse. In the future, it is expected that further research will be devoted to make progress on these topics. 

## Figures and Tables

**Table 1:** Review of the fiber optic smartphone sensors and their applications.

Ref.	Application	Smartphone	Operative System	Optical Fiber	Internal Devices	External Devices
[101]	Remote detection of hydrogen sulfide	Samsung Galaxy S6 Edge	Android 7.1.1	POF, Ø0.98/1 mm, NA 0.47	Flashlight, camera (set ISO)	3D-printed connector
[102]	Breathing pattern detection	Redmi Note 4	Android 7	POF, Ø0.98/1 mm, NA 0.47	Flashlight, camera (set ISO)	3D-printed connector
[103]	Multiplexed Breathing pattern detection	Redmi Note 4	Android 7	3xPOF, Ø0.98/1 mm, NA 0.47	Flashlight, camera (set ISO)	3D-printed connector
[104]	SPR sensor for refractive index	Huawei Ascend Y300	Android 4.1.1	Thorlabs BFL48, Ø400 μm, NA = 0.48	Flashlight, camera	Diffraction grating, couplers
[105]	Vitamin D Detection	Apple iPhone 6s	iOS	Tapered waveguide, 200–1200 μm	Flashlight, camera	Diffraction grating, external mount
[106]	Interrogation of Fabry–Perot sensor	Huawei P20 Pro	Android	Graded-index MMF, core Ø62.5 μm	Flashlight, camera	Slit, grating, 2× FC/PC connectors
[11]	Interrogation of chirped fiber Bragg grating	Huawei P20 P50	Android	Graded-index MMF, core Ø62.5 μm	Flashlight, camera	Slit, grating, 2× FC/PC connectors
[107]	Temperature Sensing			Multimode glass fiber, Ø300 μm	Flashlight, camera	Diffraction grating, pedestals, couplers
[108]	Identification of walking pattern	Motorola, Moto G3 Turbo	Android	Glass single-mode fiber	Camera	External phone mount, LED
[109]	Detection of mercuric cations	Nubia Z17 Mini	Android	Multimode glass fiber, Ø105/125 μm	Camera	Red laser, connecting module
[110]	On-site pH detection	Nubia Z17 Mini	Android	Multimode glass fiber, Ø105/125 μm	Camera	Red laser, connecting module
[111]	Dual-channel fluorescence detection	Nubia Z17 Mini	Android	Multimode glass fiber, Ø105/125 μm	Camera	Red laser, connecting module

## Abbreviations

The following abbreviations are used in this manuscript:

AMI	Acute myocardial infarction
APTMS	(3-Aminopropyl)trimethoxysilane
ARS	Alizarin Red S
B[a]P	Benzo(a)pyrene
BSA	Bovine serum albumin
CCDs	Charge-coupled devices
CD44	Cluster of differentiation 44
CIP	Ciprofloxacin
CK7	Cytokeratin 7
CK17	Cytokeratin 17
CLIA	Chemiluminescence assay
CVDs	Cardiovascular diseases
DNA	Deoxyribonucleic acid
DTC	Differentiated thyroid cancer
EBL	Electron beam lithography
ECG	Electrocardiography
EDC	*N*-ethyl-*N′*-(3-dimethylaminopropyl)carbodiimide
eFBG	Etched Fiber Bragg grating
ELISA	Enzyme-linked immunosorbent assay
ELP	Electroless deposition
eTFBG	Etched tilted Fiber Bragg grating
FWHM	Full width at half maximum
GNG	Gold nanograting
GNS	Gold nanostars
GNR	Gold nanorods
GO	Graphene oxide
GOx	Glucose oxidase
HER2	Human epidermal growth factor receptor 2
HPTS1	Hydroxy-3,6,8-pyrene trisulfonic acid trisodium salt
HTR	Human transferrin
IEMA	Immunoenzymometric assay
IRMA	Immunoradiometric assay
ITO	Indium tin oxide
LDF	Light diffusing fibers
LEDs	Light-emitting diodes
LOD	Limit-of-detection
LRSPP	Long-range surface plasmon–polariton
LSPR	Localized surface plasmon resonance
MIPs	Molecularly imprinted polymers
MMFs	Multimode fibers
MZI	Mach-Zehnder interferometer
NA	Numerical aperture
NHS	*N*-hydroxysuccinimide
NPs	Nanoparticles
OFB	Optical fiber biosensor
OFS	Optical fiber sensor
OF-SPR	Optical fiber-based surface plasmon resonance
OTA	Ochratoxin A
PANI	Polyaniline
PBS	Phosphate buffered saline
PCS	Plastic-clad silica
PDs	Photodetectors
PDMS	Polydimethylsiloxane
PMMA	Poly(methyl methacrylate)
PM-SMF	Polarization-maintaining single-mode fiber
POC	Point-of-care
POFs	Plastic optical fibers
RI	Refractive index
RIU	Refractive index units
rGO	Reduced graphene oxide
SMFs	Single-mode fibers
SNR	Signal-to-noise ratio
SPR	Surface plasmon resonance
Tn	Troponin
Tg	Thyroglobulin
TNT	Trinitrotoluene
VEGF	Vascular Endothelial Growth Factor

## References

[1] Bhalla N., Jolly P., Formisano N., Estrela P. (2016). Introduction to Biosensors. Essays Biochem..

[2] Senf B., Yeo W.-H., Kim J.-H. (2020). Recent Advances in Portable Biosensors for Biomarker Detection in Body Fluids. Biosensors.

[3] Kotsiri Z., Vidic J., Vantarakis A. (2022). Applications of Biosensors for Bacteria and Virus Detection in Food and Water–A Systematic Review. J. Environ. Sci..

[4] Ray S., Panjikar S., Anand R. (2018). Design of Protein-Based Biosensors for Selective Detection of Benzene Groups of Pollutants. ACS Sens..

[5] Chen C., Wang J. (2020). Optical Biosensors: An Exhaustive and Comprehensive Review. Analyst.

[6] Damborský P., Švitel J., Katrlík J. (2016). Optical Biosensors. Essays Biochem..

[7] Mowbray S.E., Amiri A.M. (2019). A Brief Overview of Medical Fiber Optic Biosensors and Techniques in the Modification for Enhanced Sensing Ability. Diagnostics.

[8] Sabri N., Aljunid S.A., Salim M.S., Fouad S. (2015). Fiber Optic Sensors: Short Review and Applications. Springer Series in Materials Science.

[9] Díaz C., Leitão C., Marques C., Domingues M., Alberto N., Pontes M., Frizera A., Ribeiro M., André P., Antunes P. (2017). Low-Cost Interrogation Technique for Dynamic Measurements with FBG-Based Devices. Sensors.

[10] Dos Santos P.S.S., Jorge P.A.S., de Almeida J., Coelho L. (2019). Low-Cost Interrogation System for Long-Period Fiber Gratings Applied to Remote Sensing. Sensors.

[11] Markvart A.A., Liokumovich L.B., Medvedev I.O., Ushakov N.A. (2021). Smartphone-Based Interrogation of a Chirped FBG Strain Sensor Inscribed in a Multimode Fiber. J. Light. Technol..

[12] Tien C., Lin H., Su S. (2018). High Sensitivity Refractive Index Sensor by D-Shaped Fibers and Titanium Dioxide Nanofilm. Adv. Condens. Matter Phys..

[13] Wei W., Nong J., Zhu Y., Zhang G., Wang N., Luo S., Chen N., Lan G., Chuang C.-J., Huang Y. (2018). Graphene/Au-Enhanced Plastic Clad Silica Fiber Optic Surface Plasmon Resonance Sensor. Plasmonics.

[14] Cennamo N., Chiavaioli F., Trono C., Tombelli S., Giannetti A., Baldini F., Zeni L. (2016). A Complete Optical Sensor System Based on a POF-SPR Platform and a Thermo-Stabilized Flow Cell for Biochemical Applications. Sensors.

[15] Lomer M., Arrue J., Jauregui C., Aiestaran P., Zubia J., López-Higuera J.M. (2007). Lateral Polishing of Bends in Plastic Optical Fibres Applied to a Multipoint Liquid-Level Measurement Sensor. Sensors Actuators A Phys..

[16] Perrone G., Vallan A. (2009). A Low-Cost Optical Sensor for Noncontact Vibration Measurements. IEEE Trans. Instrum. Meas..

[17] Arcas A., Dutra F., Allil R., Werneck M. (2018). Surface Plasmon Resonance and Bending Loss-Based U-Shaped Plastic Optical Fiber Biosensors. Sensors.

[18] Cennamo N., Pasquardini L., Arcadio F., Vanzetti L.E., Bossi A.M., Zeni L. (2019). D-Shaped Plastic Optical Fibre Aptasensor for Fast Thrombin Detection in Nanomolar Range. Sci. Rep..

[19] Sypabekova M., Aitkulov A., Blanc W., Tosi D. (2020). Reflector-Less Nanoparticles Doped Optical Fiber Biosensor for the Detection of Proteins: Case Thrombin. Biosens. Bioelectron..

[20] Taffoni F., Formica D., Saccomandi P., Pino G., Schena E. (2013). Optical Fiber-Based MR-Compatible Sensors for Medical Applications: An Overview. Sensors.

[21] Leitão C., Leal-Junior A., Almeida A.R., Pereira S.O., Costa F.M., Pinto J.L., Marques C. (2021). Cortisol AuPd Plasmonic Unclad POF Biosensor. Biotechnol. Rep..

[22] Gowri A., Sai V.V.R. (2016). Development of LSPR Based U-bent Plastic Optical Fiber Sensors. Sensors Actuators B Chem..

[23] Sai V.V.R., Kundu T., Mukherji S. (2009). Novel U-bent Fiber Optic Probe for Localized Surface Plasmon Resonance Based Biosensor. Biosens. Bioelectron..

[24] Corres J.M., Bravo J., Matias I.R., Arregui F.J. (2007). Tapered Optical Fiber Biosensor for the Detection of Anti-Gliadin Antibodies. Proceedings of the 2007 IEEE Sensors.

[25] Wen H.-Y., Huang C.-W., Li Y.-L., Chen J.-L., Yeh Y.-T., Chiang C.-C. (2020). A Lamping U-Shaped Fiber Biosensor Detector for MicroRNA. Sensors.

[26] Tan A.J.Y., Ng S.M., Stoddart P.R., Chua H.S. (2020). Theoretical Model and Design Considerations of U-Shaped Fiber Optic Sensors: A Review. IEEE Sens. J..

[27] Azkune M., Ruiz-Rubio L., Aldabaldetreku G., Arrospide E., Pérez-Álvarez L., Bikandi I., Zubia J., Vilas-Vilela J. (2017). U-Shaped and Surface Functionalized Polymer Optical Fiber Probe for Glucose Detection. Sensors.

[28] Yang W., Yu J., Xi X., Sun Y., Shen Y., Yue W., Zhang C., Jiang S. (2019). Preparation of Graphene/ITO Nanorod Metamaterial/U-Bent-Annealing Fiber Sensor and DNA Biomolecule Detection. Nanomaterials.

[29] George A., Amrutha M.S., Srivastava P., Sunil S., Sai V.V.R., Srinivasan R. (2021). Development of a U-bent Plastic Optical Fiber Biosensor with Plasmonic Labels for the Detection of Chikungunya Non-Structural Protein 3. Analyst.

[30] Gowri A., Rajamani A.S., Ramakrishna B., Sai V.V.R. (2019). U-bent Plastic Optical Fiber Probes as Refractive Index Based Fat Sensor for Milk Quality Monitoring. Opt. Fiber Technol..

[31] Divagar M., Gowri A., John S., Sai V.V.R. (2018). Graphene Oxide Coated U-bent Plastic Optical Fiber Based Chemical Sensor for Organic Solvents. Sensors Actuators B Chem..

[32] Chen K.-C., Li Y.-L., Wu C.-W., Chiang C.-C. (2018). Glucose Sensor Using U-Shaped Optical Fiber Probe with Gold Nanoparticles and Glucose Oxidase. Sensors.

[33] Bandaru R., Divagar M., Khanna S., Danny C.G., Gupta S., Janakiraman V., Sai V.V.R. (2020). U-bent Fiber Optic Plasmonic Biosensor Platform for Ultrasensitive Analyte Detection. Sensors Actuators B Chem..

[34] Manoharan H., Kalita P., Gupta S., Sai V.V.R. (2019). Plasmonic Biosensors for Bacterial Endotoxin Detection on Biomimetic C-18 Supported Fiber Optic Probes. Biosens. Bioelectron..

[35] Lu P., Men L., Sooley K., Chen Q. (2009). Tapered Fiber Mach–Zehnder Interferometer for Simultaneous Measurement of Refractive Index and Temperature. Appl. Phys. Lett..

[36] Ayupova T., Shaimerdenova M., Tosi D. (2021). Shallow-Tapered Chirped Fiber Bragg Grating Sensors for Dual Refractive Index and Temperature Sensing. Sensors.

[37] Latifi H., Zibaii M.I., Hosseini S.M., Jorge P. (2012). Nonadiabatic Tapered Optical Fiber for Biosensor Applications. Photonic Sens..

[38] Dash S.P., Patnaik S.K., Tripathy S.K. (2019). Investigation of a Low Cost Tapered Plastic Fiber Optic Biosensor Based on Manipulation of Colloidal Gold Nanoparticles. Opt. Commun..

[39] Lin H.-Y., Huang C.-H., Cheng G.-L., Chen N.-K., Chui H.-C. (2012). Tapered Optical Fiber Sensor Based on Localized Surface Plasmon Resonance. Opt. Express.

[40] Rahman H.A., Harun S.W., Yasin M., Phang S.W., Damanhuri S.S.A., Arof H., Ahmad H. (2011). Tapered Plastic Multimode Fiber Sensor for Salinity Detection. Sens. Actuators A Phys..

[41] (2019). Pesavento; Profumo; Merli; Cucca; Zeni; Cennamo An Optical Fiber Chemical Sensor for the Detection of Copper(II) in Drinking Water. Sensors.

[42] Zheng H., Huang B., Li Y., Zhang R., Gu X., Li Z., Lin H., Zhu W., Tang J., Guan H. (2020). Residual Thickness Enhanced Core-Removed D-Shaped Single-Mode Fiber and Its Application for VOC Evaporation Monitoring. Opt. Express.

[43] Sequeira F., Cennamo N., Rudnitskaya A., Nogueira R., Zeni L., Bilro L. (2019). D-Shaped POF Sensors for Refractive Index Sensing—The Importance of Surface Roughness. Sensors.

[44] Ying Y., Si G., Luan F., Xu K., Qi Y., Li H. (2017). Recent Research Progress of Optical Fiber Sensors Based on D-Shaped Structure. Opt. Laser Technol..

[45] Cennamo N., D’Agostino G., Pasquardini L., Arcadio F., Perri C., Coppola N., Angelillo I.F., Altucci L., Di Marzo F., Parisio E.M. (2021). (INVITED)Quantitative Detection of SARS-CoV-2 Virions in Aqueous Mediums by IoT Optical Fiber Sensors. Results Opt..

[46] Cennamo N., Pesavento M., Zeni L. (2021). A Review on Simple and Highly Sensitive Plastic Optical Fiber Probes for Bio-Chemical Sensing. Sens. Actuators B Chem..

[47] Cennamo N., Donà A., Pallavicini P., D’Agostino G., Dacarro G., Zeni L., Pesavento M. (2015). Sensitive Detection of 2,4,6-Trinitrotoluene by Tridimensional Monitoring of Molecularly Imprinted Polymer with Optical Fiber and Five-Branched Gold Nanostars. Sens. Actuators B Chem..

[48] Cennamo N., D’Agostino G., Perri C., Arcadio F., Chiaretti G., Parisio E.M., Camarlinghi G., Vettori C., Di Marzo F., Cennamo R. (2021). Proof of Concept for a Quick and Highly Sensitive On-Site Detection of SARS-CoV-2 by Plasmonic Optical Fibers and Molecularly Imprinted Polymers. Sensors.

[49] Pasquardini L., Cennamo N., Malleo G., Vanzetti L., Zeni L., Bonamini D., Salvia R., Bassi C., Bossi A.M. (2021). A Surface Plasmon Resonance Plastic Optical Fiber Biosensor for the Detection of Pancreatic Amylase in Surgically-Placed Drain Effluent. Sensors.

[50] Loyez M., Lobry M., Hassan E.M., DeRosa M.C., Caucheteur C., Wattiez R. (2021). HER2 Breast Cancer Biomarker Detection Using a Sandwich Optical Fiber Assay. Talanta.

[51] Ortega-Gomez A., Barroso J., Calatayud-Sánchez A., Zubia J., Benito-Lopez F., Basabe-Desmonts L., Villatoro J. (2021). Cytochrome c Detection by Plasmonic Nanospectroscopy on Optical Fiber Facets. Sensors Actuators B Chem..

[52] Xiong Y., Xu F. (2020). Multifunctional Integration on Optical Fiber Tips: Challenges and Opportunities. Adv. Photonics.

[53] Lee B., Park J.-H., Byun J.-Y., Kim J.H., Kim M.-G. (2018). An Optical Fiber-Based LSPR Aptasensor for Simple and Rapid in-Situ Detection of Ochratoxin A. Biosens. Bioelectron..

[54] Righini G., Soria S. (2016). Biosensing by WGM Microspherical Resonators. Sensors.

[55] Xu X., Chen W., Zhao G., Li Y., Lu C., Yang L. (2018). Wireless Whispering-Gallery-Mode Sensor for Thermal Sensing and Aerial Mapping. Light Sci. Appl..

[56] Chiasera A., Dumeige Y., Féron P., Ferrari M., Jestin Y., Nunzi Conti G., Pelli S., Soria S., Righini G.C. (2010). Spherical Whispering-Gallery-Mode Microresonators. Laser Photon. Rev..

[57] Shaimerdenova M., Ayupova T., Sypabekova M., Tosi D. (2020). Fiber Optic Refractive Index Sensors Based on a Ball Resonator and Optical Backscatter Interrogation. Sensors.

[58] Sypabekova M., Korganbayev S., González-Vila Á., Caucheteur C., Shaimerdenova M., Ayupova T., Bekmurzayeva A., Vangelista L., Tosi D. (2019). Functionalized Etched Tilted Fiber Bragg Grating Aptasensor for Label-Free Protein Detection. Biosens. Bioelectron..

[59] Bekmurzayeva A., Dukenbayev K., Shaimerdenova M., Bekniyazov I., Ayupova T., Sypabekova M., Molardi C., Tosi D. (2018). Etched Fiber Bragg Grating Biosensor Functionalized with Aptamers for Detection of Thrombin. Sensors.

[60] Aitkulov A., Sypabekova M., Molardi C., Blanc W., Tosi D. (2021). Fabrication and Performance Evaluation of Reflectorless Refractive Index Fiber Optic Sensors Using Etched Enhanced Backscattering Fibers. Measurement.

[61] Cennamo N., Trigona C., Graziani S., Zeni L., Arcadio F., Xiaoyan L., Di Pasquale G., Pollicino A. (2021). Green LSPR Sensors Based on Thin Bacterial Cellulose Waveguides for Disposable Biosensor Implementation. IEEE Trans. Instrum. Meas..

[62] Cennamo N., Saitta L., Tosto C., Arcadio F., Zeni L., Fragalá M.E., Cicala G. (2021). Microstructured Surface Plasmon Resonance Sensor Based on Inkjet 3D Printing Using Photocurable Resins with Tailored Refractive Index. Polymers.

[63] Arcadio F., Zeni L., Montemurro D., Eramo C., Di Ronza S., Perri C., D’Agostino G., Chiaretti G., Porto G., Cennamo N. (2021). Biochemical Sensing Exploiting Plasmonic Sensors Based on Gold Nanogratings and Polymer Optical Fibers. Photonics Res..

[64] Zeni L., Pesavento M., Marchetti S., Cennamo N. (2018). [INVITED] Slab Plasmonic Platforms Combined with Plastic Optical Fibers and Molecularly Imprinted Polymers for Chemical Sensing. Opt. Laser Technol..

[65] Cennamo N., Pasquardini L., Arcadio F., Lunelli L., Vanzetti L., Carafa V., Altucci L., Zeni L. (2021). SARS-CoV-2 Spike Protein Detection through a Plasmonic D-Shaped Plastic Optical Fiber Aptasensor. Talanta.

[66] Cennamo N., Varriale A., Pennacchio A., Staiano M., Massarotti D., Zeni L., D’Auria S. (2013). An Innovative Plastic Optical Fiber-Based Biosensor for New Bio/Applications. The Case of Celiac Disease. Sens. Actuators B Chem..

[67] Cennamo N., Mattiello F., Zeni L. (2017). Slab Waveguide and Optical Fibers for Novel Plasmonic Sensor Configurations. Sensors.

[68] Arcadio F., Zeni L., Perri C., D’Agostino G., Chiaretti G., Porto G., Minardo A., Cennamo N. (2021). Bovine Serum Albumin Protein Detection by a Removable SPR Chip Combined with a Specific MIP Receptor. Chemosensors.

[69] Arcadio F., Zeni L., Minardo A., Eramo C., Di Ronza S., Perri C., D’Agostino G., Chiaretti G., Porto G., Cennamo N. (2021). A Nanoplasmonic-Based Biosensing Approach for Wide-Range and Highly Sensitive Detection of Chemicals. Nanomaterials.

[70] Cennamo N., Zeni L., Catalano E., Arcadio F., Minardo A. (2018). Refractive Index Sensing through Surface Plasmon Resonance in Light-Diffusing Fibers. Appl. Sci..

[71] Cennamo N., Zeni L., Arcadio F., Catalano E., Minardo A. (2019). A Novel Approach to Realizing Low-Cost Plasmonic Optical Fiber Sensors: Light-Diffusing Fibers Covered by Thin Metal Films. Fibers.

[72] Cennamo N., Arcadio F., Prete D.D., Buonanno G., Minardo A., Pirozzi S., Zeni L. (2021). A Simple and Efficient Plasmonic Sensor in Light Diffusive Polymer Fibers. IEEE Sens. J..

[73] Cennamo N., Trono C., Giannetti A., Baldini F., Minardo A., Zeni L., Tombelli S. (2021). Biosensors Exploiting Unconventional Platforms: The Case of Plasmonic Light-Diffusing Fibers. Sens. Actuators B Chem..

[74] Cennamo N., Arcadio F., Zeni L., Catalano E., Del Prete D., Buonanno G., Minardo A. (2021). The Role of Tapered Light-Diffusing Fibers in Plasmonic Sensor Configurations. Sensors.

[75] Cennamo N., Coelho L., Santos D.F., Baptista J.M., Guerreiro A., Jorge P.A.S., Zeni L. (2015). Modal Filtering for Optimized Surface Plasmon Resonance Sensing in Multimode Plastic Optical Fibers. IEEE Sens. J..

[76] Tosi D., Schena E., Molardi C., Korganbayev S. (2018). Fiber Optic Sensors for Sub-Centimeter Spatially Resolved Measurements: Review and Biomedical Applications. Opt. Fiber Technol..

[77] Issatayeva A., Beisenova A., Tosi D., Molardi C. (2020). Fiber-Optic Based Smart Textiles for Real-Time Monitoring of Breathing Rate. Sensors.

[78] Guo T., González-Vila Á., Loyez M., Caucheteur C. (2017). Plasmonic Optical Fiber-Grating Immunosensing: A Review. Sensors.

[79] Poeggel S., Tosi D., Duraibabu D., Leen G., McGrath D., Lewis E. (2015). Optical Fibre Pressure Sensors in Medical Applications. Sensors.

[80] Liao K.-C., Chang S.-C., Chiu C.-Y., Chou Y.-H. (2010). Acute Response in Vivo of a Fiber-Optic Sensor for Continuous Glucose Monitoring from Canine Studies on Point Accuracy. Sensors.

[81] Issatayeva A., Amantayeva A., Blanc W., Tosi D., Molardi C. (2021). Design and Analysis of a Fiber-Optic Sensing System for Shape Reconstruction of a Minimally Invasive Surgical Needle. Sci. Rep..

[82] Loyez M., Larrieu J.-C., Chevineau S., Remmelink M., Leduc D., Bondue B., Lambert P., Devière J., Wattiez R., Caucheteur C. (2019). In Situ Cancer Diagnosis through Online Plasmonics. Biosens. Bioelectron..

[83] Poeggel S., Duraibabu D., Tosi D., Leen G., Lewis E., McGrath D., Fusco F., Sannino S., Lupoli L., Ippolito J. (2015). Differential in Vivo Urodynamic Measurement in a Single Thin Catheter Based on Two Optical Fiber Pressure Sensors. J. Biomed. Opt..

[84] Guo T., Liu F., Liang X., Qiu X., Huang Y., Xie C., Xu P., Mao W., Guan B.-O., Albert J. (2016). Highly Sensitive Detection of Urinary Protein Variations Using Tilted Fiber Grating Sensors with Plasmonic Nanocoatings. Biosens. Bioelectron..

[85] Liao K.-C., Hogen-Esch T., Richmond F.J., Marcu L., Clifton W., Loeb G.E. (2008). Percutaneous Fiber-Optic Sensor for Chronic Glucose Monitoring in Vivo. Biosens. Bioelectron..

[86] Beisenova A., Issatayeva A., Iordachita I., Blanc W., Molardi C., Tosi D. (2019). Distributed Fiber Optics 3D Shape Sensing by Means of High Scattering NP-Doped Fibers Simultaneous Spatial Multiplexing. Opt. Express.

[87] Rantala J., Hännikäinen J., Vanhala J. (2011). Fiber Optic Sensors for Wearable Applications. Pers. Ubiquitous Comput..

[88] Lo Presti D., Massaroni C., D’Abbraccio J., Massari L., Caponero M., Longo U.G., Formica D., Oddo C.M., Schena E. (2019). Wearable System Based on Flexible FBG for Respiratory and Cardiac Monitoring. IEEE Sens. J..

[89] Li H., Yang H., Li E., Liu Z., Wei K. (2012). Wearable Sensors in Intelligent Clothing for Measuring Human Body Temperature Based on Optical Fiber Bragg Grating. Opt. Express.

[90] Esmaeilzadeh H., Rivard M., Arzi E., Légaré F., Hassani A. (2015). Smart Textile Plasmonic Fiber Dew Sensors. Opt. Express.

[91] Merazzo K.J., Totoricaguena-Gorriño J., Fernández-Martín E., del Campo F.J., Baldrich E. (2021). Smartphone-Enabled Personalized Diagnostics: Current Status and Future Prospects. Diagnostics.

[92] de Haan K., Ceylan Koydemir H., Rivenson Y., Tseng D., Van Dyne E., Bakic L., Karinca D., Liang K., Ilango M., Gumustekin E. (2020). Automated Screening of Sickle Cells Using a Smartphone-Based Microscope and Deep Learning. NPJ Digit. Med..

[93] Hernández-Neuta I., Neumann F., Brightmeyer J., Ba Tis T., Madaboosi N., Wei Q., Ozcan A., Nilsson M. (2019). Smartphone-based Clinical Diagnostics: Towards Democratization of Evidence-based Health Care. J. Intern. Med..

[94] McCracken K.E., Tat T., Paz V., Yoon J.-Y. (2017). Smartphone-Based Fluorescence Detection of Bisphenol A from Water Samples. RSC Adv..

[95] Severi C., Melnychuk N., Klymchenko A.S. (2020). Smartphone-Assisted Detection of Nucleic Acids by Light-Harvesting FRET-Based Nanoprobe. Biosens. Bioelectron..

[96] Khachornsakkul K., Dungchai W. (2020). Development of an Ultrasound-Enhanced Smartphone Colorimetric Biosensor for Ultrasensitive Hydrogen Peroxide Detection and Its Applications. RSC Adv..

[97] Morosanova M.A., Bashkatova A.S., Morosanova E.I. (2019). Spectrophotometric and Smartphone-Assisted Determination of Phenolic Compounds Using Crude Eggplant Extract. Molecules.

[98] Geng Z., Zhang X., Fan Z., Lv X., Su Y., Chen H. (2017). Recent Progress in Optical Biosensors Based on Smartphone Platforms. Sensors.

[99] Hussain I., Das M., Ahamad K.U., Nath P. (2017). Water Salinity Detection Using a Smartphone. Sens. Actuators B Chem..

[100] Saini S.S., Sridhar A., Ahluwalia K. (2019). Smartphone Optical Sensors. Opt. Photonics News.

[101] Sultangazin A., Kusmangaliyev J., Aitkulov A., Akilbekova D., Olivero M., Tosi D. (2017). Design of a Smartphone Plastic Optical Fiber Chemical Sensor for Hydrogen Sulfide Detection. IEEE Sens. J..

[102] Aitkulov A., Tosi D. (2019). Optical Fiber Sensor Based on Plastic Optical Fiber and Smartphone for Measurement of the Breathing Rate. IEEE Sens. J..

[103] Aitkulov A., Tosi D. (2019). Design of an All-POF-Fiber Smartphone Multichannel Breathing Sensor with Camera-Division Multiplexing. IEEE Sensors Lett..

[104] Bremer K., Roth B. (2015). Fibre Optic Surface Plasmon Resonance Sensor System Designed for Smartphones. Opt. Express.

[105] Walter J.-G., Alwis L.S.M., Roth B., Bremer K. (2020). All-Optical Planar Polymer Waveguide-Based Biosensor Chip Designed for Smartphone-Assisted Detection of Vitamin D. Sensors.

[106] Markvart A., Liokumovich L., Medvedev I., Ushakov N. (2020). Continuous Hue-Based Self-Calibration of a Smartphone Spectrometer Applied to Optical Fiber Fabry-Perot Sensor Interrogation. Sensors.

[107] Pan T., Cao W., Wang M. (2018). TiO2 Thin Film Temperature Sensor Monitored by Smartphone. Opt. Fiber Technol..

[108] Kamizi M.A., Negri L.H., Fabris J.L., Muller M. (2019). A Smartphone Based Fiber Sensor for Recognizing Walking Patterns. IEEE Sens. J..

[109] Liu T., Wang W., Jian D., Li J., Ding H., Yi D., Liu F., Wang S. (2019). Quantitative Remote and On-Site Hg2+ Detection Using the Handheld Smartphone Based Optical Fiber Fluorescence Sensor (SOFFS). Sens. Actuators B Chem..

[110] Liu T., Wang W., Ding H., Yi D. (2019). Smartphone-Based Hand-Held Optical Fiber Fluorescence Sensor for On-Site PH Detection. IEEE Sens. J..

[111] Liu T., Wang W., Ding H., Liu Z., Zhang S., Yi D. (2020). Development of a Handheld Dual-Channel Optical Fiber Fluorescence Sensor Based on a Smartphone. Appl. Opt..

[112] Peltomaa R., Glahn-Martínez B., Benito-Peña E., Moreno-Bondi M. (2018). Optical Biosensors for Label-Free Detection of Small Molecules. Sensors.

[113] Yu B., Huang Y., Zhou J., Guo T., Guan B.-O. (2016). Understanding the PH-Dependent Interaction between Graphene Oxide and Single-Stranded DNA through a Fiber-Optic Interferometer. Phys. Chem. Chem. Phys..

[114] Aebersold R., Anderson L., Caprioli R., Druker B., Hartwell L., Smith R. (2005). Perspective: A Program to Improve Protein Biomarker Discovery for Cancer. J. Proteome Res..

[115] Chen X.-H., Huang S., Kerr D. (2011). Biomarkers in Clinical Medicine. IARC Sci. Publ..

[116] Mass R.D., Press M.F., Anderson S., Cobleigh M.A., Vogel C.L., Dybdal N., Leiberman G., Slamon D.J. (2005). Evaluation of Clinical Outcomes According to HER2 Detection by Fluorescence In Situ Hybridization in Women with Metastatic Breast Cancer Treated with Trastuzumab. Clin. Breast Cancer.

[117] Martinez P., Hernández-Losa J., Cedrés S., Castellví J., Martinez-Marti A., Tallada N., Murtra-Garrell N., Navarro-Mendivill A., Rodriguez-Freixinos V., Canela M. (2013). Fluorescence In Situ Hybridization and Immunohistochemistry as Diagnostic Methods for ALK Positive Non-Small Cell Lung Cancer Patients. PLoS ONE.

[118] Sieben V.J., Debes Marun C.S., Pilarski P.M., Kaigala G.V., Pilarski L.M., Backhouse C.J. (2007). FISH and Chips: Chromosomal Analysis on Microfluidic Platforms. IET Nanobiotechnol..

[119] Ramos-Vara J.A. (2005). Technical Aspects of Immunohistochemistry. Vet. Pathol..

[120] Mukundan H., Kubicek J.Z., Holt A., Shively J.E., Martinez J.S., Grace K., Grace W.K., Swanson B.I. (2009). Planar Optical Waveguide-Based Biosensor for the Quantitative Detection of Tumor Markers. Sens. Actuators B Chem..

[121] Ribaut C., Loyez M., Larrieu J.-C., Chevineau S., Lambert P., Remmelink M., Wattiez R., Caucheteur C. (2017). Cancer Biomarker Sensing Using Packaged Plasmonic Optical Fiber Gratings: Towards in Vivo Diagnosis. Biosens. Bioelectron..

[122] Loyez M., Lobry M., Wattiez R., Caucheteur C. (2019). Optical Fiber Gratings Immunoassays. Sensors.

[123] Ribaut C., Voisin V., Malachovská V., Dubois V., Mégret P., Wattiez R., Caucheteur C. (2016). Small Biomolecule Immunosensing with Plasmonic Optical Fiber Grating Sensor. Biosens. Bioelectron..

[124] Sun D., Ran Y., Wang G. (2017). Label-Free Detection of Cancer Biomarkers Using an In-Line Taper Fiber-Optic Interferometer and a Fiber Bragg Grating. Sensors.

[125] Ross J.S., Fletcher J.A., Linette G.P., Stec J., Clark E., Ayers M., Symmans W.F., Pusztai L., Bloom K.J. (2003). The HER-2/ Neu Gene and Protein in Breast Cancer 2003: Biomarker and Target of Therapy. Oncologist.

[126] Doi T., Shitara K., Naito Y., Shimomura A., Fujiwara Y., Yonemori K., Shimizu C., Shimoi T., Kuboki Y., Matsubara N. (2017). Safety, Pharmacokinetics, and Antitumour Activity of Trastuzumab Deruxtecan (DS-8201), a HER2-Targeting Antibody–Drug Conjugate, in Patients with Advanced Breast and Gastric or Gastro-Oesophageal Tumours: A Phase 1 Dose-Escalation Study. Lancet Oncol..

[127] Basakran N.S. (2015). CD44 as a Potential Diagnostic Tumor Marker. Saudi Med. J..

[128] Ristamäki R., Joensuu H., Jalkanen S. (1999). Serum CD44 in Non-Hodgkin’s Lymphoma. Leuk. Lymphoma.

[129] Wang S.J., Wong G., de Heer A.-M., Xia W., Bourguignon L.Y.W. (2009). CD44 Variant Isoforms in Head and Neck Squamous Cell Carcinoma Progression. Laryngoscope.

[130] Yan Y., Zuo X., Wei D. (2015). Concise Review: Emerging Role of CD44 in Cancer Stem Cells: A Promising Biomarker and Therapeutic Target. Stem Cells Transl. Med..

[131] Bekmurzayeva A., Ashikbayeva Z., Myrkhiyeva Z., Nugmanova A., Shaimerdenova M., Ayupova T., Tosi D. (2021). Label-Free Fiber-Optic Spherical Tip Biosensor to Enable Picomolar-Level Detection of CD44 Protein. Sci. Rep..

[132] Davies L., Welch H.G. (2014). Current Thyroid Cancer Trends in the United States. JAMA Otolaryngol. Neck Surg..

[133] Benbassat C.A., Mechlis-Frish S., Guttmann H., Glaser B., Krausz Y. (2007). Current Concepts in the Follow-up of Patients with Differentiated Thyroid Cancer. Isr. Med. Assoc. J..

[134] McLeod D.S.A. (2010). Current Concepts and Future Directions in Differentiated Thyroid Cancer. Clin. Biochem. Rev..

[135] Eustatia-Rutten C.F.A., Smit J.W.A., Romijn J.A., van der Kleij-Corssmit E.P.M., Pereira A.M., Stokkel M.P., Kievit J. (2004). Diagnostic Value of Serum Thyroglobulin Measurements in the Follow-up of Differentiated Thyroid Carcinoma, a Structured Meta-Analysis. Clin. Endocrinol..

[136] Pellegriti G., Frasca F., Regalbuto C., Squatrito S., Vigneri R. (2013). Worldwide Increasing Incidence of Thyroid Cancer: Update on Epidemiology and Risk Factors. J. Cancer Epidemiol..

[137] Giovanella L., Clark P.M., Chiovato L., Duntas L., Elisei R., Feldt-Rasmussen U., Leenhardt L., Luster M., Schalin-Jäntti C., Schott M. (2014). DIAGNOSIS OF ENDOCRINE DISEASE: Thyroglobulin Measurement Using Highly Sensitive Assays in Patients with Differentiated Thyroid Cancer: A Clinical Position Paper. Eur. J. Endocrinol..

[138] Kim H.-M., Jeong D.H., Lee H.-Y., Park J.-H., Lee S.-K. (2021). Design and Validation of Fiber Optic Localized Surface Plasmon Resonance Sensor for Thyroglobulin Immunoassay with High Sensitivity and Rapid Detection. Sci. Rep..

[139] Man Y., Cao J., Jin S., Xu G., Pan B., Shang L., Che D., Yu Q., Yu Y. (2014). Newly Identified Biomarkers for Detecting Circulating Tumor Cells in Lung Adenocarcinoma. Tohoku J. Exp. Med..

[140] Sharma S.V., Bell D.W., Settleman J., Haber D.A. (2007). Epidermal Growth Factor Receptor Mutations in Lung Cancer. Nat. Rev. Cancer.

[141] Schiller J.H., Harrington D., Belani C.P., Langer C., Sandler A., Krook J., Zhu J., Johnson D.H. (2002). Comparison of Four Chemotherapy Regimens for Advanced Non–Small-Cell Lung Cancer. N. Engl. J. Med..

[142] Omary M.B., Ku N.O.N. (1997). Intermediate Filament Proteins of the Liver: Emerging Disease Association and Functions. Hepatology.

[143] Chu P.G., Weiss L.M. (2002). Keratin Expression in Human Tissues and Neoplasms. Histopathology.

[144] DePianto D., Kerns M.L., Dlugosz A.A., Coulombe P.A. (2010). Keratin 17 Promotes Epithelial Proliferation and Tumor Growth by Polarizing the Immune Response in Skin. Nat. Genet..

[145] WHO (2019). World Health Statistics 2019: Monitoring Health for the SDGs, Sustainable Development Goals.

[146] Piepoli M.F., Hoes A.W., Agewall S., Albus C., Brotons C., Catapano A.L., Cooney M.-T., Corrà U., Cosyns B., Deaton C. (2016). 2016 European Guidelines on Cardiovascular Disease Prevention in Clinical Practice. Eur. Heart J..

[147] Visseren F.L.J., Mach F., Smulders Y.M., Carballo D., Koskinas K.C., Bäck M., Benetos A., Biffi A., Boavida J.-M., Capodanno D. (2021). 2021 ESC Guidelines on Cardiovascular Disease Prevention in Clinical Practice. Eur. Heart J..

[148] Ouyang M., Tu D., Tong L., Sarwar M., Bhimaraj A., Li C., Coté G.L., Di Carlo D. (2021). A Review of Biosensor Technologies for Blood Biomarkers toward Monitoring Cardiovascular Diseases at the Point-of-Care. Biosens. Bioelectron..

[149] Leitão C., Ribau V., Afreixo V., Antunes P., André P., Pinto J.L., Boutouyrie P., Laurent S., Bastos J.M. (2018). Clinical Evaluation of an Optical Fiber-Based Probe for the Assessment of Central Arterial Pulse Waves. Hypertens. Res..

[150] Kumar S., Singh R., Kaushik B.K., Chen N., Yang Q.S., Zhang X. (2019). LSPR-Based Cholesterol Biosensor Using Hollow Core Fiber Structure. IEEE Sens. J..

[151] Zhou W., Li K., Wei Y., Hao P., Chi M., Liu Y., Wu Y. (2018). Ultrasensitive Label-Free Optical Microfiber Coupler Biosensor for Detection of Cardiac Troponin I Based on Interference Turning Point Effect. Biosens. Bioelectron..

[152] Krupin O., Berini P. (2019). Long-Range Surface Plasmon-Polariton Waveguide Biosensors for Human Cardiac Troponin I Detection. Sensors.

[153] Liu T., Liang L.-L., Xiao P., Sun L.-P., Huang Y.-Y., Ran Y., Jin L., Guan B.-O. (2018). A Label-Free Cardiac Biomarker Immunosensor Based on Phase-Shifted Microfiber Bragg Grating. Biosens. Bioelectron..

[154] Botewad S.N., Pahurkar V.G., Muley G.G., Gaikwad D.K., Bodkhe G.A., Shirsat M.D., Pawar P.P. (2020). PANI-ZnO Cladding-Modified Optical Fiber Biosensor for Urea Sensing Based on Evanescent Wave Absorption. Front. Mater..

[155] Yang Q., Zhang X., Kumar S., Singh R., Zhang B., Bai C., Pu X. (2020). Development of Glucose Sensor Using Gold Nanoparticles and Glucose-Oxidase Functionalized Tapered Fiber Structure. Plasmonics.

[156] Zheng W., Han B., Siyu E., Sun Y., Li X., Cai Y., Zhang Y. (2020). Highly-Sensitive and Reflective Glucose Sensor Based on Optical Fiber Surface Plasmon Resonance. Microchem. J..

[157] Jeremias A. (2010). The Utility of Troponin Measurement to Detect Myocardial Infarction: Review of the Current Findings. Vasc. Health Risk Manag..

[158] Boeddinghaus J., Reichlin T., Nestelberger T., Twerenbold R., Meili Y., Wildi K., Hillinger P., Giménez M.R., Cupa J., Schumacher L. (2017). Early Diagnosis of Acute Myocardial Infarction in Patients with Mild Elevations of Cardiac Troponin. Clin. Res. Cardiol..

[159] Al-Naher A., Wright D., Devonald M.A.J., Pirmohamed M. (2018). Renal Function Monitoring in Heart Failure-What Is the Optimal Frequency? A Narrative Review. Br. J. Clin. Pharmacol..

[160] Ferguson M.A., Waikar S.S. (2012). Established and Emerging Markers of Kidney Function. Clin. Chem..

[161] Li M., Singh R., Marques C., Zhang B., Kumar S. (2021). 2D Material Assisted SMF-MCF-MMF-SMF Based LSPR Sensor for Creatinine Detection. Opt. Express.

[162] Vogt S., Ruppert V., Pankuweit S., Paletta J.P.J., Rhiel A., Weber P., Irqsusi M., Cybulski P., Ramzan R. (2018). Myocardial Insufficiency Is Related to Reduced Subunit 4 Content of Cytochrome c Oxidase. J. Cardiothorac. Surg..

[163] Dar T., Radfar A., Abohashem S., Pitman R.K., Tawakol A., Osborne M.T. (2019). Psychosocial Stress and Cardiovascular Disease. Curr. Treat. Options Cardiovasc. Med..

[164] Osborne M.T., Shin L.M., Mehta N.N., Pitman R.K., Fayad Z.A., Tawakol A. (2020). Disentangling the Links between Psychosocial Stress and Cardiovascular Disease. Circ. Cardiovasc. Imaging.

[165] Pulopulos M.M., Vanderhasselt M.-A., De Raedt R. (2018). Association between Changes in Heart Rate Variability during the Anticipation of a Stressful Situation and the Stress-Induced Cortisol Response. Psychoneuroendocrinology.

[166] Usha S.P., Gupta B.D. (2015). Fiber Optic SPR Based P-Cresol Sensor Using Ag/ZnO Nanoparticle-Chitosan/Tyrosinanse. Proceedings of the JSAP-OSA Joint Symposia 2015.

[167] Gupta V. (2002). Removal of Lindane and Malathion from Wastewater Using Bagasse Fly Ash—a Sugar Industry Waste. Water Res..

[168] Gupta V.K., Tyagi I., Agarwal S., Singh R., Chaudhary M., Harit A., Kushwaha S. (2016). Column Operation Studies for the Removal of Dyes and Phenols Using a Low Cost Adsorbent. Glob. J. Environ. Sci. Manag..

[169] Michałowicz J., Duda W. (2007). Phenols—Sources and Toxicity. Polish J. Environ. Stud..

[170] King R.A., May B.L., Davies D.A., Bird A.R. (2009). Measurement of Phenol and P-Cresol in Urine and Feces Using Vacuum Microdistillation and High-Performance Liquid Chromatography. Anal. Biochem..

[171] Usha S.P., Gupta B.D. (2018). Urinary P-Cresol Diagnosis Using Nanocomposite of ZnO/MoS2 and Molecular Imprinted Polymer on Optical Fiber Based Lossy Mode Resonance Sensor. Biosens. Bioelectron..

[172] Li Y.-F., Liu Z.-M., Liu Y.-L., Yang Y.-H., Shen G.-L., Yu R.-Q. (2006). A Mediator-Free Phenol Biosensor Based on Immobilizing Tyrosinase to ZnO Nanoparticles. Anal. Biochem..

[173] Adamski J., Nowak P., Kochana J. (2010). Simple Sensor for the Determination of Phenol and Its Derivatives in Water Based on Enzyme Tyrosinase. Electrochim. Acta.

[174] Wang Y., Zhu G., Li M., Singh R., Marques C., Min R., Kaushik B.K., Zhang B., Jha R., Kumar S. (2021). Water Pollutants P-Cresol Detection Based on Au-ZnO Nanoparticles Modified Tapered Optical Fiber. IEEE Trans. Nanobioscience.

[175] Cennamo N., Zeni L., Ricca E., Isticato R., Marzullo V.M., Capo A., Staiano M., D’Auria S., Varriale A. (2019). Detection of Naphthalene in Sea-Water by a Label-Free Plasmonic Optical Fiber Biosensor. Talanta.

[176] Lamarca R.S., Franco D.F., Nalin M., de Lima Gomes P.C.F., Messaddeq Y. (2020). Label-Free Ultrasensitive and Environment-Friendly Immunosensor Based on a Silica Optical Fiber for the Determination of Ciprofloxacin in Wastewater Samples. Anal. Chem..

[177] Gao R., Lu D.-F., Zhang M.-Y., Qi Z.-M. (2018). Optofluidic Immunosensor Based on Resonant Wavelength Shift of a Hollow Core Fiber for Ultratrace Detection of Carcinogenic Benzo[a]Pyrene. ACS Photonics.

[178] González-Vila Á., Debliquy M., Lahem D., Zhang C., Mégret P., Caucheteur C. (2017). Molecularly Imprinted Electropolymerization on a Metal-Coated Optical Fiber for Gas Sensing Applications. Sensors Actuators B Chem..

[179] Ke Z.-J., Tang D.-L., Lai X., Dai Z.-Y., Zhang Q. (2018). Optical Fiber Evanescent-Wave Sensing Technology of Hydrogen Sulfide Gas Concentration in Oil and Gas Fields. Optik.

[180] Prado A.R., Díaz C.A.R., Lyra Nunes L.G., Oliveira J.P., Guimarães M.C.C., Leal-Junior A., Ribeiro M.R.N., Pontes M.J. (2021). Surface Plasmon Resonance-Based Optical Fiber Sensors for H2S In Situ Detection. Plasmonics.

[181] Chu C.-S., Lo Y.-L. (2008). Fiber-Optic Carbon Dioxide Sensor Based on Fluorinated Xerogels Doped with HPTS. Sens. Actuators B Chem..

[182] Shanavas S., Ahamad T., Alshehri S.M., Acevedo R., Anbarasan P.M. (2021). A Facile Microwave Route for Fabrication of NiO/RGO Hybrid Sensor with Efficient CO2 and Acetone Gas Sensing Performance Using Clad Modified Fiber Optic Method. Optik.

[183] Allsop T., Arif R., Neal R., Kalli K., Kundrát V., Rozhin A., Culverhouse P., Webb D.J. (2016). Photonic Gas Sensors Exploiting Directly the Optical Properties of Hybrid Carbon Nanotube Localized Surface Plasmon Structures. Light Sci. Appl..

[184] Pinto V., Sousa P., Catarino S.O., Correia-Neves M., Minas G. (2017). Microfluidic Immunosensor for Rapid and Highly-Sensitive Salivary Cortisol Quantification. Biosens. Bioelectron..

[185] Holsboer F., Ising M. (2010). Stress Hormone Regulation: Biological Role and Translation into Therapy. Annu. Rev. Psychol..

[186] Usha S.P., Shrivastav A.M., Gupta B.D. (2017). A Contemporary Approach for Design and Characterization of Fiber-Optic-Cortisol Sensor Tailoring LMR and ZnO/PPY Molecularly Imprinted Film. Biosens. Bioelectron..

[187] Sharma A.K., Kaur B., Marques C. (2020). Simulation and Analysis of 2D Material/Metal Carbide Based Fiber Optic SPR Probe for Ultrasensitive Cortisol Detection. Optik.

[188] Randall D., Tsui T.K. (2002). Ammonia Toxicity in Fish. Mar. Pollut. Bull..

[189] Zhu Y., Fu H., Ding J., Li H., Zhang M., Zhang J., Liu Y. (2018). Fabrication of Three-Dimensional Zinc Oxide Nanoflowers for High-Sensitivity Fiber-Optic Ammonia Gas Sensors. Appl. Opt..

[190] Shrivastav A.M., Sharma G., Rathore A.S., Jha R. (2018). Hypersensitive and Selective Interferometric Nose for Ultratrace Ammonia Detection with Fast Response Utilizing PANI@SnO 2 Nanocomposite. ACS Photonics.

[191] Leal-Junior A.G., Frizera A., Marques C. (2020). Low-Cost Fiberoptic Probe for Ammonia Early Detection in Fish Farms. Remote Sens..

[192] Miliutina E., Guselnikova O., Burtsev V., Elashnikov R., Postnikov P., Svorcik V., Lyutakov O. (2020). Plasmon-Active Optical Fiber Functionalized by Metal Organic Framework for Pesticide Detection. Talanta.

[193] Kant R. (2020). Surface Plasmon Resonance Based Fiber–Optic Nanosensor for the Pesticide Fenitrothion Utilizing Ta2O5 Nanostructures Sequestered onto a Reduced Graphene Oxide Matrix. Microchim. Acta.

